# Reanalyzing DNA mixture: a evaluation of EuroForMix for deconvolution and weight-of-evidence computing

**DOI:** 10.1007/s12024-024-00872-x

**Published:** 2025-10-02

**Authors:** Aline Costa Minervino, Cristina Moniz de Aragão Gualda, Bruno Rodrigues Trindade, Carlos Eduardo Martinez de Medeiros, Ronaldo Carneiro da Silva Júnior

**Affiliations:** 1 Department of Public and Forensic Health Sciences, and Medical Education of the, University of Porto, Porto, Portugal; 2 Brazilian National DNA Database, Federal Police, SPO Quadra 7 Lote 23, Brasília-DF, 70610-200 Brazil; 3 National Police Academy, Federal Police, Brasília-DF, Brazil; 4 National Institute of Criminalistics, Federal Police, Brasília-DF, Brazil

**Keywords:** DNA interpretation, Likelihood Ratio, Continuous method, Quantitative Model, Forensic genetics

## Abstract

Forensic genetics faces significant challenges in the evolving landscape of DNA mixture analysis. This paper highlights the complexities associated with stochastic effects and artifacts in degraded or low-quantity samples and explores the primary objectives of DNA mixture analysis, namely deconvolution and weight of evidence quantification. The study examines the efficacy of the software tool EuroForMix (EFM) in interpreting complex mixtures. Genetic profiles from two forensic cases processed in 2022 by the Brazilian National Institute of Criminalistics' DNA Forensic Laboratory were reanalyzed using EFM v.3.4.0, focusing on deconvoluting DNA mixtures and quantifying the weight of evidence. Results were compared with previous analyses conducted using a laboratory-validated spreadsheet, LRmix Studio (for computing LR), and GeneMapperTM ID-X (for deconvoluting mixtures). EFM demonstrated high efficiency in both deconvolution and weight-of-evidence quantification, showing improved LR values for various profiles compared to previous analyses. In the reanalyzed cases, weight of evidence calculations using EFM produced values comparable to those obtained with the laboratory-validated spreadsheet and superior LR values compared to LRmix Studio. The comparison of deconvoluted profiles using EFM and GeneMapperTM ID-X revealed mostly consistent results for the major contributor genotype, with EFM yielding equal or better outcomes in most profiles. Thus, EFM shows potential as a tool for DNA mixture analysis, including both LR computation and deconvolution. Despite these encouraging results, it is recommended that each forensic laboratory develop DNA mixture interpretation protocols that consider internal validation.

**Supplementary Information:**

The online version contains supplementary material available at 10.1007/s12024-024-00872-x.

## Introduction

DNA analysis has become a fundamental tool in forensic science for identifying individuals. However, when crime scene samples contain DNA from multiple individuals, interpreting these mixtures can be particularly challenging. The primary goals of DNA mixture analysis are to deconvolute the individual profiles within the mixture and/or to quantify the weight of evidence regarding the presence of specific individuals. Complicating this process are stochastic effects and artifacts often encountered in degraded or low-quantity samples.

Forensic genetics has significantly advanced criminal investigations by providing critical evidence for solving crimes. Crime scene samples may contain DNA from both the victim and potential perpetrators, making it crucial to accurately determine the sources of DNA stains for effective criminal investigation [1]. The interpretation of DNA profiles, especially in the context of mixtures, is a complex process requiring expertise in both molecular biology and statistical analysis [2].

A DNA mixture sample is defined as a biological sample originating from two or more donors, indicated by the presence of three or more alleles or unbalanced peak heights at the same locus [3]. The challenge in interpreting such mixtures lies in quantifying the likelihood of genotype combinations contributed by the different individuals. Determining the number of contributors is particularly difficult when DNA in the mixture is highly degraded, when the contributors’ proportions are unequal, or when the number of contributors is unknown [4]. Additionally, low-quality and low-quantity DNA samples are especially prone to stochastic effects and artifacts.

Over the past thirty years, forensic genetic techniques have evolved significantly. Advances in DNA extraction methods, multiplex STR amplification, capillary electrophoresis instrumentation, and the use of increased PCR cycles have enhanced the sensitivity of these methods [3]. However, this increased sensitivity also results in more complex and challenging DNA mixtures [4, 5]. While earlier forensic analyses primarily dealt with high-quality DNA samples, current forensic laboratories frequently handle lower quality and quantity samples, including mixtures from multiple donors, which complicates DNA profile interpretation [3, 4].

Earlier, DNA mixtures were relatively rare. For instance, in a 2003 review by Torres et al., which analyzed 1,547 criminal cases from various Spanish regions over four years (1997–2000), only 163 out of 2,424 samples (6.7%) showed mixed profiles [6]. The study highlighted that each mixed sample analysis could be multifactorial and challenging to interpret [6].In recent years, forensic laboratories have increasingly analyzed trace evidence from various sources, including swabs from handguns and items related to property crimes. A 2016 study by Chemale et al. reported on over 4,200 biological samples from 1,072 property crime cases processed by the Brazilian Federal Police Forensic Genetics Laboratory between 2011 and 2016 [7]. The study found that the majority of biological material recovered was trace/contact DNA (45.8%), followed by material from worn clothing (22.8%). This led to the analysis of numerous mixture profiles from these trace samples, which often contained low-quality or low-quantity DNA and presented significant challenges due to complex mixtures and low-level contributors [4].

The objectives of DNA mixture analysis include both the deconvolution of the mixture profile and the quantification of evidence weight. Deconvolution aims to estimate the genotypes of the mixture's contributors, either by identifying major and minor contributors or by inferring the profile of an unknown contributor from known alleles [3, 8]. The deduced profiles are then compared with other case profiles or DNA database entries [1, 8]. The weight of evidence is typically expressed using statistical principles, commonly through Likelihood Ratios (LRs). Various statistical models have been developed to quantify the weight of evidence in DNA mixtures, though these models can be complex and necessitate specialized software for practical application. The reliability of these analyses depends on the biological, mathematical, statistical, and informatics models used [9].

In this study, we reanalyze a selection of forensic mixtures. Previously, weight of evidence was quantified using LRmix Studio or a laboratory-validated spreadsheet, and deconvolutions were performed using GeneMapper™ ID-X. In the current study, we utilize the EuroForMix (EFM) software for both LR calculations and deconvolutions.

## Material and methods

Genetic profiles from two forensic cases processed in 2022 by the DNA Forensic Laboratory of the Brazilian National Institute of Criminalistics were reanalyzed. These cases were selected due to the presence of crime stains that matched reference samples. Initially, Likelihood Ratios (LRs) for these cases were calculated using LRmix Studio 2.1.3 or a laboratory-validated spreadsheet, while DNA mixtures were deconvoluted using GeneMapper™ ID-X 1.6.

In the present study, the genetic profiles were reanalyzed using EFM v.3.4.0 to perform mixture deconvolution and quantify the weight of evidence. The following settings were utilized: Easy mode set to “NO,” default detection threshold at “50,” default FST-correction at “0.02” [10], default probability of drop-in at “0.0005,” default drop-in hyperparameter at “0.01,” and both prior BW and FW stutter-proportion functions set to “dbeta(x,1,1)” [11]. The maximum number of loci considered was “30.” Allelic frequencies used were those recommended for the Brazilian National DNA Database [10], except for the SE33 marker, which was excluded from LR computations. The STR amplification was performed using the PowerPlex® Fusion 6C kit. The analytical thresholds for each marker were determined based on internal validation parameters for each dye color (in RFU: TMR-117, CXR-123, TOM-123, JOE-178, FL-135) [12]. Both degradation models and stutter effects (backward and forward) were taken into account for the analysis.

The weight-of-evidence was quantified using the “Optimal Quantitative LR” model. The significance level for model validation was set at 0.01, and the Maximum Likelihood Estimate (MLE) for model parameters was reported. The number of non-contributors was set to 100, and the number of Markov Chain Monte Carlo (MCMC) sample iterations was set to 10,000. For deconvolution, the major contributor genotype was predicted using the Top Marginal Table estimation under Hd with a probability greater than 95%.

## The experiment

### Case 1

On June 10, 2022, the DNA Forensic Laboratory received five crime stains collected on May 27, 2022, from the rear compartment of a vehicle involved in a police incident that resulted in a civilian’s death in Sergipe, Brazil. The crime stains included swabs with blood or biological traces from the floor of the rear compartment: vestige 4 (sample Q1), vestige 5 (sample Q2), vestige 6 (sample Q3), vestige 7 (sample Q4), and vestige 9 (sample Q5). Additionally, a reference sample (ref1), consisting of the victim’s blood on an FTA card®, was also received.

Analysis of samples Q2 and Q3 identified two distinct male individuals, yielding unique genetic profiles. Samples Q1, Q4, and Q5 each resulted in DNA mixture profiles involving at least two contributors. From the reference sample ref1, a male-specific genetic profile was obtained. Notably, unique profile from Q3 matched the profile of ref1, leading to the use of a laboratory-validated spreadsheet to calculate the weight of evidence for this match. Moreover, shared alleles were found between the profile of ref1 and the minority component of the DNA mixture profile from Q1, so the weight of evidence for Q1 was computed using LRmix Studio.

The five crime stains (Q1-Q5) and the reference sample (ref1) were imported into EFM for analysis. Each crime stain was plotted based on allele and peak height, with ref1 labeled as sample 1. Analysis revealed that the minority component of the DNA mixture in Q1 shared alleles with ref1, and the unitary profile from Q3 matched ref1.

Model validation for crime stains Q1 and Q3 was performed using both the Hd and Hp models, with a significance level set at 0.01. The weight of evidence for these samples was computed using EFM, and the results were compared with those from previous analyses (Table 1). The cumulative distribution of LR values for 100 non-contributors replacing ref1 with an LR greater than 1 were 0.02 for Q1 and 0.00 for Q3.

**Table 1:** Pairwise Comparison of LR Values from Prior and Current Analyses

Sample Comparison	Prior Analysis LR	Reanalysis LR (EFM)
Q1 and ref1	6.969e + 13 (LRmix Studio)	1.465e + 23
Q3 and ref1	2.309e + 30 (spreadsheet)	2.288e + 30

Deconvolution of the DNA mixtures Q4 and Q5, which were analyzed as having two unknown contributors, was performed using EFM. The major contributor profiles obtained from EFM were compared with those from prior analyses using GeneMapper™ ID-X (Fig. 1). No discrepancies were found between the results of the two software tools, except for the FGA locus in Q4, which was inconclusive using the ID-X tool.

### Case 2

On May 17, 2022, the DNA Forensic Laboratory received thirty-two crime samples collected on March 25, 2022, from vehicles abandoned after police confrontations on Highway BR-277 in Paraná, Brazil. This study focused on several crime stains: a blood swab from vehicle 1 (Q1), a blood-soaked towel from vehicle 1 (Q3), a blood swab from vehicle 2 (Q6), a white coat from vehicle 2 (Q7), a blood-soaked tissue from vehicle 3 (Q15), a swab from the steering wheel of vehicle 3 (Q18), a barbecue stick from vehicle 3 (Q19), and a cigarette butt from vehicle 3 (Q29). Additionally, biological samples were collected from two suspects, ref2 and ref3, via buccal swabbing.

The analysis revealed that DNA mixtures Q1, Q7, Q18, and Q19 shared alleles with ref2, while unitary profiles Q3 and Q6 matched ref1. Moreover, DNA mixture Q15 and unitary profile Q29 shared alleles with ref3. In prior analyses, the weight of evidence was calculated using a laboratory-validated spreadsheet for most samples, except for Q29, which was analyzed using LRmix Studio. Mixture profiles were initially deconvoluted using GeneMapper™ ID-X to identify the major component.

The eight crime stains (Q1, Q3, Q6, Q7, Q15, Q18, Q19, and Q29) and the two reference samples (ref2 and ref3) were imported into EFM for analysis. Each crime stain was plotted based on allele and peak height.

Model validation for all eight crime stains was conducted using both the Hd and Hp models, with a significance level of 0.01. The weights of evidence were computed using EFM and compared with the previous results (Table 2). The cumulative distribution of LR values for 100 non-contributors replacing ref2 with an LR greater than 1 was 0.00 for Q1, Q3, Q6, Q7, Q18, and Q19. For ref3, the cumulative distribution of LR values greater than 1 was 0.02 for Q15 and 0.00 for Q29.

**Table 2:** Pairwise Comparison of LR Values from Prior and Current Analyses

Sample Comparison	Prior Analysis LR	Reanalysis LR (EFM)
Q1 and ref2	3.593e + 10 (spreadsheet)	2.789e + 21
Q3 and ref2	2.755e + 26 (spreadsheet)	2.755e + 26
Q6 and ref2	2.755e + 26 (spreadsheet)	2.755e + 26
Q7 and ref2	2.380e + 21 (spreadsheet)	2.449e + 26
Q18 and ref2	5.268e + 20 (spreadsheet)	3.205e + 26
Q19 and ref2	5.851e + 14 (spreadsheet)	3.374e + 26
Q15 and ref3	6.341e + 10 (spreadsheet)	5.459e + 22
Q29 and ref3	2.198e + 25 (LRmix Studio)	6.214e + 26

Deconvolution of the DNA mixtures (Q1, Q7, Q18, Q19, and Q15) was performed using EuroForMix. The major contributor profiles obtained were compared with those from previous analyses using GeneMapper™ ID-X. Both software tools provided consistent results in most cases, except for some loci where one tool was inconclusive:

- Case2Q7: EFM deconvolution was inconclusive for loci D22S1045 and TPOX, while ID-X was inconclusive for loci D12S391, D18S51, D3S1358, and TH01.
- Case2Q18: EFM was conclusive for all autosomal loci, whereas ID-X was inconclusive for loci D12S391, D16S539, D18S51, and D1S1656.
- Case2Q19: EFM was conclusive for all autosomal loci, while ID-X was inconclusive for loci D16S539, D18S51, D19S433, D1S1656, D2S1338, TH01, VWA, D7S820, and TPOX.
- Case2Q15: Both EFM and ID-X were inconclusive for several loci, including D12S391, D18S51, D19S433, D1S1656, D22S1045, D2S1338, CSF1PO, D13S317, D5S818, D7S820, TPOX, and Penta D. EFM was also inconclusive for D2S441 and Penta E, while ID-X was inconclusive for FGA and VWA (Fig. 2).

## Discussion

Recent advancements in statistical methods have addressed many challenges in DNA mixture analysis, enhancing the accuracy and reliability of interpreting complex DNA profiles. These new approaches represent significant progress beyond earlier techniques, which often relied on subjective evaluation by practitioners. However, implementing these advanced methods can be complex and necessitate specialized statistical expertise. Consequently, the development of specialized software aims to facilitate the use of these advanced statistical approaches, aiding in the interpretation of DNA mixtures and the estimation of the weight of evidence.

Quantitative models, while more complex, offer a more refined analysis by reducing subjectivity in DNA typing results. These models are sensitive to artifacts and stochastic effects but provide powerful tools for including contributors and excluding non-contributors from DNA profiles [9, 13, 14]. Several software tools based on quantitative models have been developed to assist experts by considering various parameters such as population co-ancestry coefficients, allelic frequencies, detection thresholds, drop-in distributions, and stutter modeling [4, 15].

Studies comparing results from different statistical software have shown that depending on the tool used, outcomes can vary significantly by several orders of magnitude. Evaluating the sensitivity and specificity of these software tools is crucial for understanding their applicability in forensic casework. The performance of studies that test the sensitivity and specificity results of statistical software plays a fundamental role in constantly evaluating the application of models in different situations that may occur in forensic casework [4, 16].

The primary objective of this study was to reanalyze two DNA mixture cases previously examined at the Federal Police forensic genetics laboratory and compare the results. Initially, these cases were analyzed using LRmix Studio, GeneMapper™ ID-X, and a laboratory-validated spreadsheet. The reanalysis aimed to streamline the expert analysis process by adopting EFM for both deconvolution and weight of evidence quantification.

In the reanalyzed cases, EFM provided weight of evidence calculations for unitary profiles (Case1Q3, Case2Q3, and Case2Q6) that were consistent with the initial results obtained using the laboratory-validated spreadsheet. EFM was preferred over the spreadsheet due to its ability to import evidence files and reduce the inaccuracies associated with manual data entry.

For the two cases analyzed with LRmix Studio—one involving a mixture profile (Case1Q1) and the other a unitary profile with allele drop-out (Case2Q29)—EFM produced higher LR values compared to those from LRmix Studio (1.465e + 23 vs. 6.969e + 13 for Case1Q1|ref1, and 6.214e + 26 vs. 2.198e + 25 for Case2Q29|ref3).

For other mixture profiles (Case2Q1, Case2Q7, Case2Q15, Case2Q18, and Case2Q19), where initial analyses used GeneMapper™ ID-X for deconvolution and a laboratory-validated spreadsheet for LR calculations, EFM provided significantly higher LR values (e.g., 2.789e + 21 vs. 3.593e + 10 for Case2Q1|ref2, 2.449e + 26 vs. 2.380e + 21 for Case2Q7|ref2). These differences are attributed to the more effective deconvolution analysis performed by EFM.

The comparison of deconvoluted profiles from EFM and GeneMapper™ ID-X generally showed agreement in the major contributor genotype. However, some markers were not reported by one software or the other. EFM either matched or exceeded the number of loci assigned to the major contributor compared to GeneMapper™ ID-X, except for Case2Q1, where EFM could not determine the major genotype. EFM uses specific hypotheses, such as Hd, to estimate the posterior probability of the combined genotype [17, 18]. In this study, the top marginal deconvolution method was employed to identify the most likely genotype for each contributor. Mixtures with near-equal contributions often yielded less optimal results than those with more unbalanced contributions [19]. In Case2Q1, the equal contribution of 50% from each contributor may have led to inconclusive genotype results.

ITwo areas for improvement in EFM were identified: incorporating amelogenin deconvolution and including essential audit data in the deconvolution report, such as sample codes and model specifications. Despite these areas for improvement, EFM provided consistent results with the initial analysis while offering a faster, more straightforward process and reduced risk of manual data entry errors.

Other studies using EFM have suggested that the height of genotype probability may indicate the true genotype, though occasionally high probabilities corresponded with incorrect genotypes. They suggested that the ratio to the next genotype value may offer a more practical selection method for confidently determining the major contributor's genotype [19]. Further research is needed to validate EFM's effectiveness in deconvolving mixture profiles.

While the software provides valuable quantitative measures of the weight of evidence that assist forensic analysts in interpreting complex DNA mixtures, it cannot replace the need for properly qualified practitioners. Researchers agree that while statistical methodologies and software are essential and should be encouraged, the ultimate responsibility for critically evaluating and accurately interpreting DNA mixture results lies with the analyst [3, 6, 14]. The expertise of the analyst is crucial to ensuring the correct application of these tools and the accurate interpretation of the data.

## Conclusion

Forensic DNA mixture interpretation has become an essential aspect of criminal investigations. DNA mixtures from crime scenes present significant challenges, especially when the samples contain minimal or degraded DNA, exhibit unequal contributions from multiple individuals, or involve an unknown number of contributors. Advanced statistical methods and specialized software have been developed to enhance the accuracy and reliability of interpreting such complex mixtures, which are common in cases such as sexual assaults or property crimes. Variability in results among different statistical tools highlights the necessity for rigorous evaluation and testing of sensitivity and specificity in forensic casework.

EuroForMix has demonstrated its potential as an effective tool for analyzing DNA mixtures, offering improvements in both the calculation of likelihood ratios (LR) and deconvolution processes. In this study, the use of EFM produced results that were consistent with those from the laboratory-validated spreadsheet for weight of evidence calculations. Moreover, LR values computed using EFM showed notable improvements compared to those obtained with LRmix Studio. For deconvolution, EFM and GeneMapper™ ID-X generally yielded comparable results, though some discrepancies were noted for markers not considered or inconclusive in one of the software.

The reanalysis of the two cases using EFM confirmed the findings from the initial analysis, with some results showing enhanced LR values and more comprehensive deconvolution of major contributor loci. EFM provides a streamlined approach by integrating both deconvolution and LR calculation into a single software solution. It considers various factors such as co-ancestry coefficients, population allele frequencies, and rates of drop-out, drop-in, and degradation.

The primary focus of this case report was to evaluate the performance of EFM and to compare it with previously used methods. The results indicate that EFM shows promising improvements in these areas. However, this evaluation is still ongoing, and additional studies, including statistical tests, are necessary to confirm EFM's reliability and effectiveness across various scenarios. The limited sample size of this retrospective study restricts the generalizability of the results. Despite these limitations, our findings provide valuable insights into the potential benefits of using EFM for DNA mixture analysis.

Despite these promising results, it is crucial to acknowledge the limitations of the software and the need for expert interpretation. Forensic laboratories should develop and adhere to robust DNA mixture interpretation protocols, incorporating internal validation, allele frequency data, and specific software parameters. Practitioners must be thoroughly trained in statistical methodologies, understanding both the limitations of the technology and the potential for error. Staying informed about the latest research and best practices in forensic genetics is essential to prevent misinterpretations.

Clear and transparent reporting of DNA mixture results is vital to maintain the reliability and credibility of forensic evidence. This includes detailed explanations of the methods and assumptions used for interpretation and presenting results in a clear and concise manner. Such practices help avoid misunderstandings and ensure that the evidence is accurately considered in the context of the case.Ongoing research and development are crucial for advancing DNA mixture interpretation techniques and enhancing the accuracy and reliability of forensic genetics. Improved methods will contribute to establishing a more robust weight of evidence for evaluation by stakeholders, including investigators, lawyers, and judges, thereby supporting well-informed decision-making.

## Key points

I) Challenges in DNA mixtures: The paper addresses the complexities of analyzing crime stains, particularly due to stochastic effects and artifacts present in degraded or low-quantity samples.

II) Objectives of DNA Mixture Analysis: Key objectives include the deconvolution of DNA mixtures and the quantification of the weight of evidence, which are critical for accurate forensic analysis.

III) Software Evaluation: The study evaluates EuroForMix (EFM) version 3.4.0 as a software tool for interpreting complex DNA mixtures, demonstrating its efficiency in deconvolution and weight-of-evidence quantification.

IV) Comparison of Analysis Results: Reanalysis of two forensic cases revealed that EuroForMix (EFM) provided improved likelihood ratio (LR) values compared to previous analyses, suggesting EFM's potential for forensic applications.

## Supplementary Information

Below is the link to the electronic supplementary material.

Supplementary file1 (DOCX 6236 KB)

Supplementary file2 (DOCX 6236 KB)

## Data Availability

The analyzed data originates from real criminal cases, being restricted access to the Brazilian Federal Police.

## Declarations

Ethical approval
