# Supplementary material for: Reanalyzing DNA mixture: a evaluation of EuroForMix for deconvolution and weight-of-evidence computing
Source: Forensic Sci Med Pathol. 2025 Oct 2;21(4):1813–9. doi: 10.1007/s12024-024-00872-x (PMC12799628; doi:10.1007/s12024-024-00872-x)
Supplement: Supplementary file 1 — Supplementary file1 (DOCX 6236 KB) [file 12024_2024_872_MOESM1_ESM.docx]

SUPPLEMENT

Case 1

|  |
| --- |
| Figure 1. Crime scene forensic analyses photo taken by Deitos, A.R. on May 27, 2022 |

| CASE1  Q1  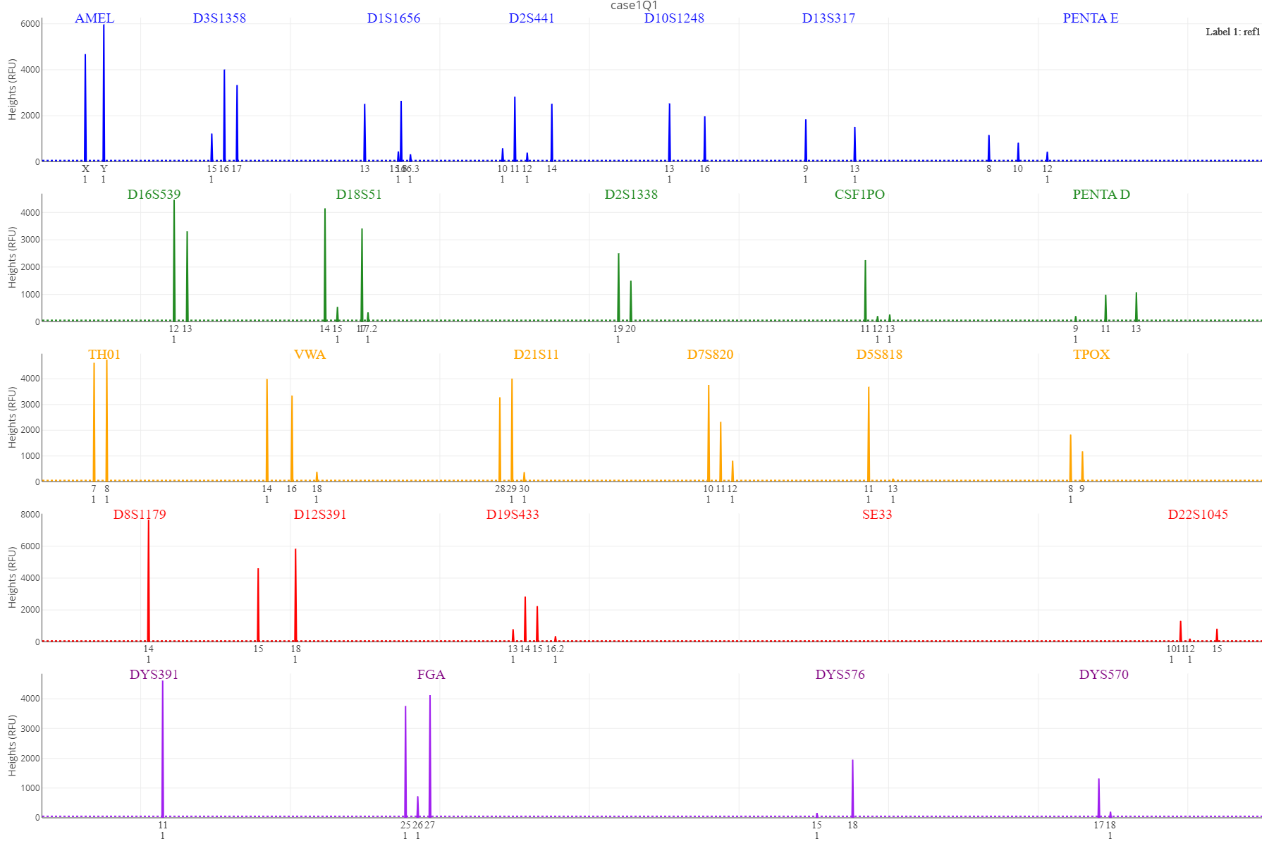 |
| --- |
| Q2  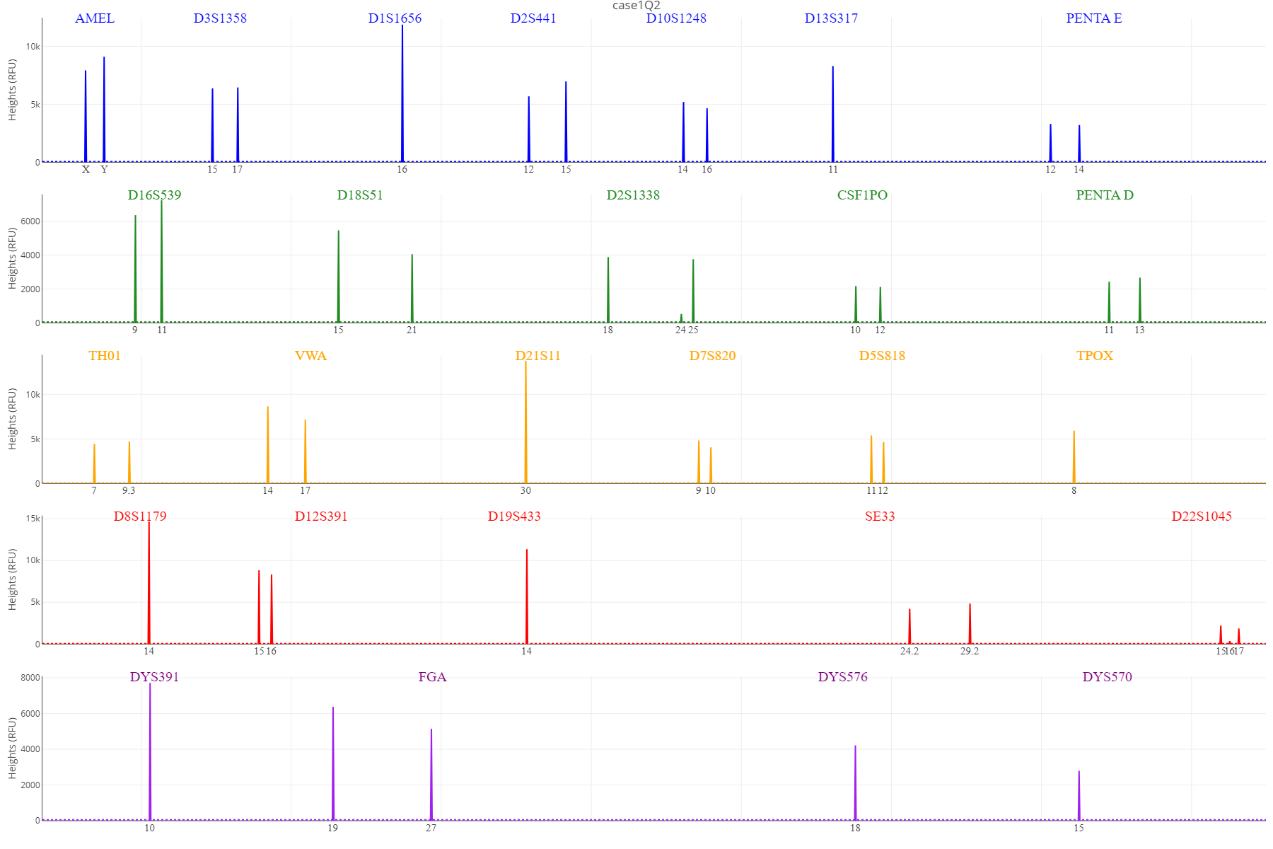 |
| Q3  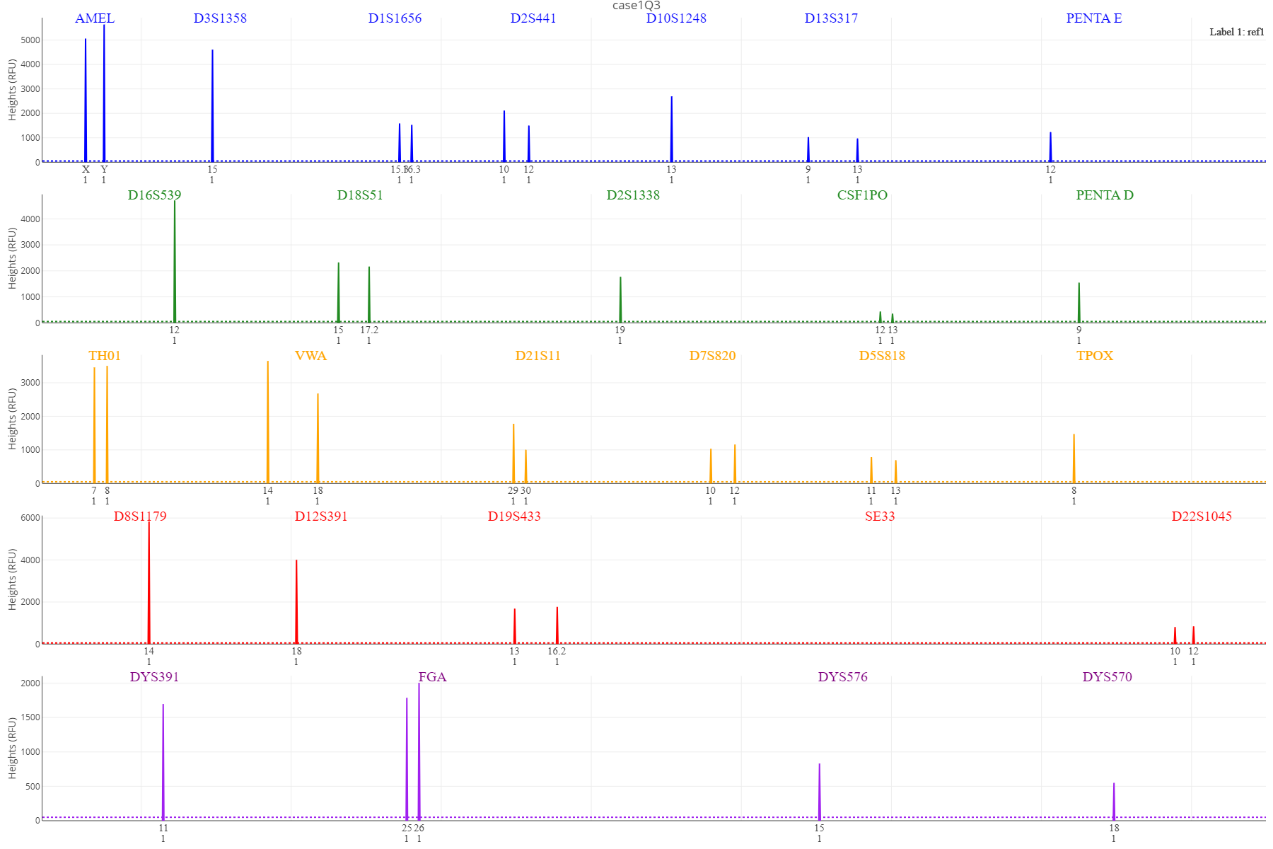 |
| Q4  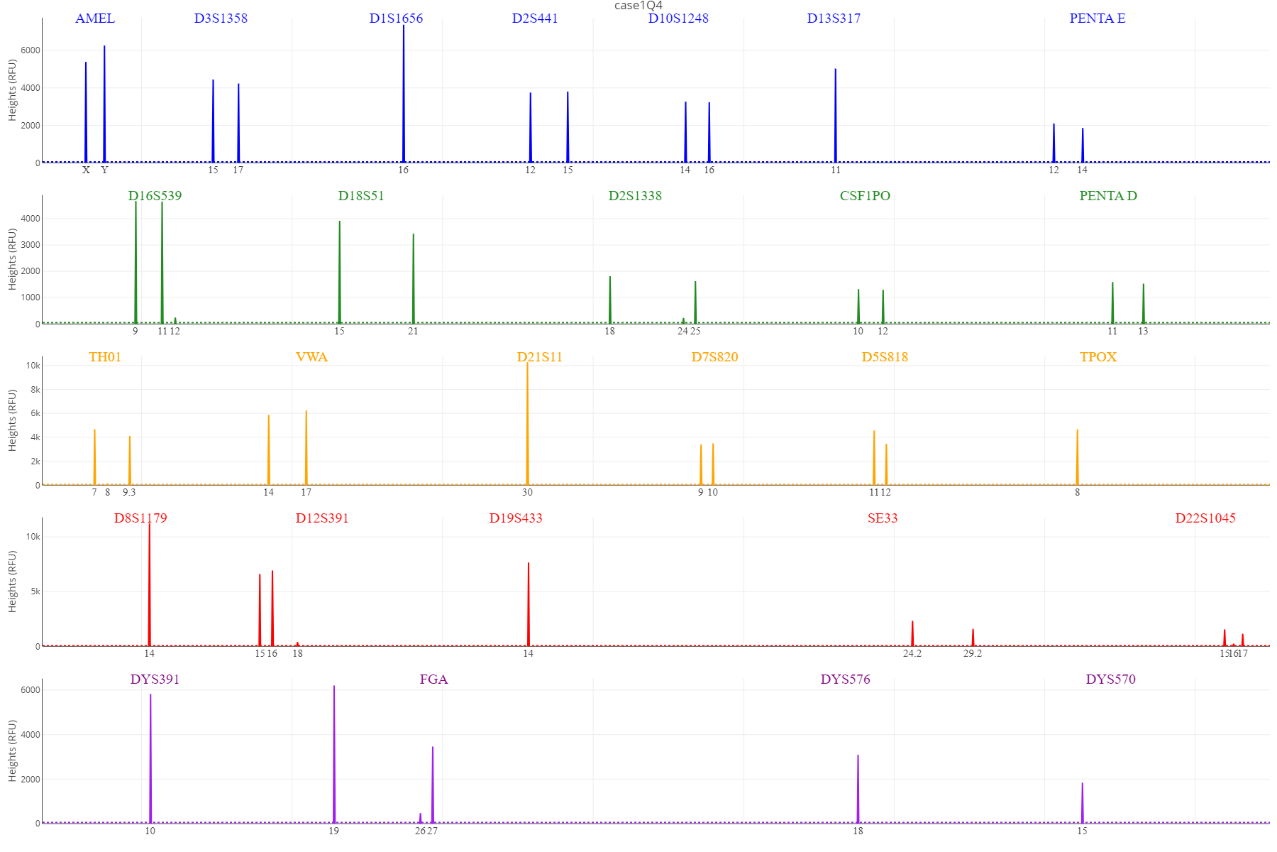 |
| Q5  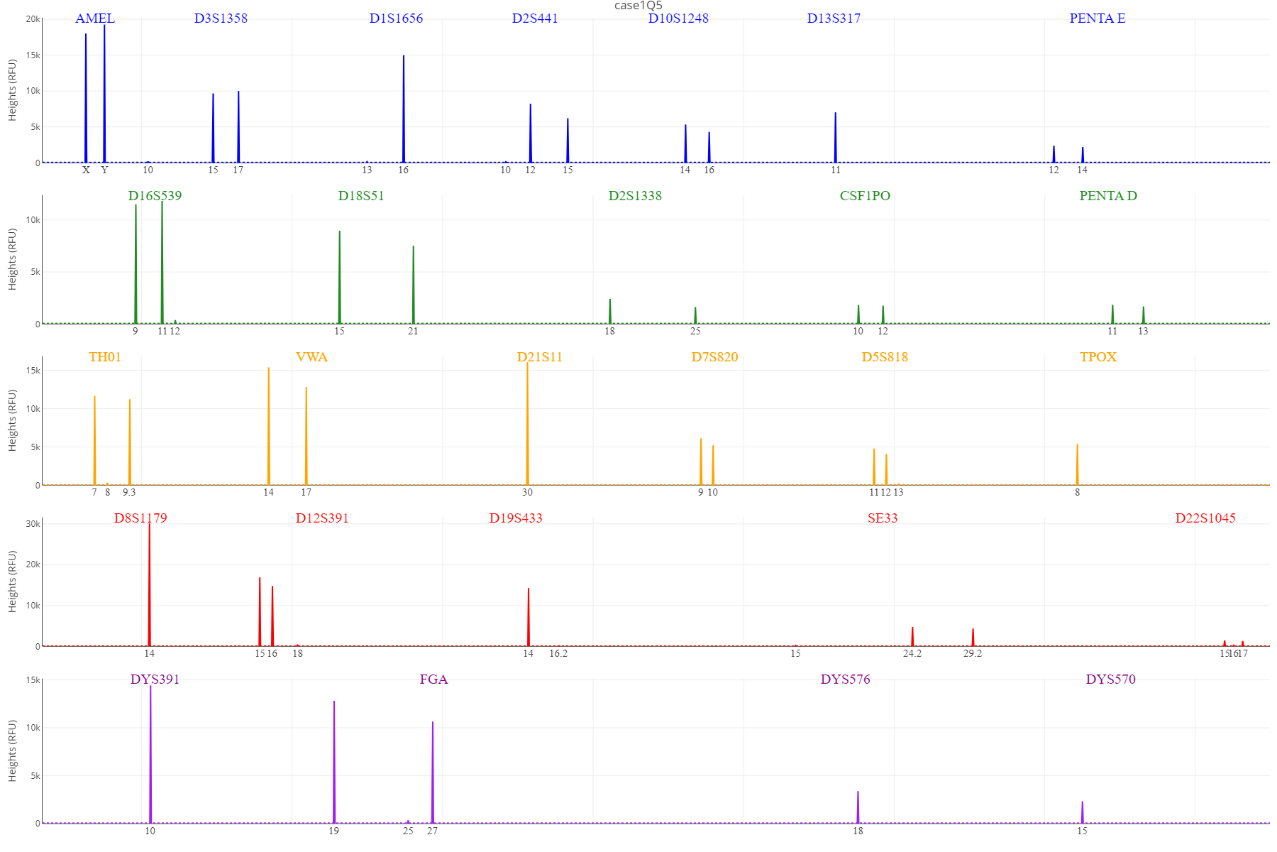 |
| Figure 2. The allele and peak height representations of Case 1 crime stains Q1, Q2, Q3, Q4, and Q5 are shown. The reference ref1 is labeled with the number "1" in Q1 and Q3. |

| CASE1  Q1  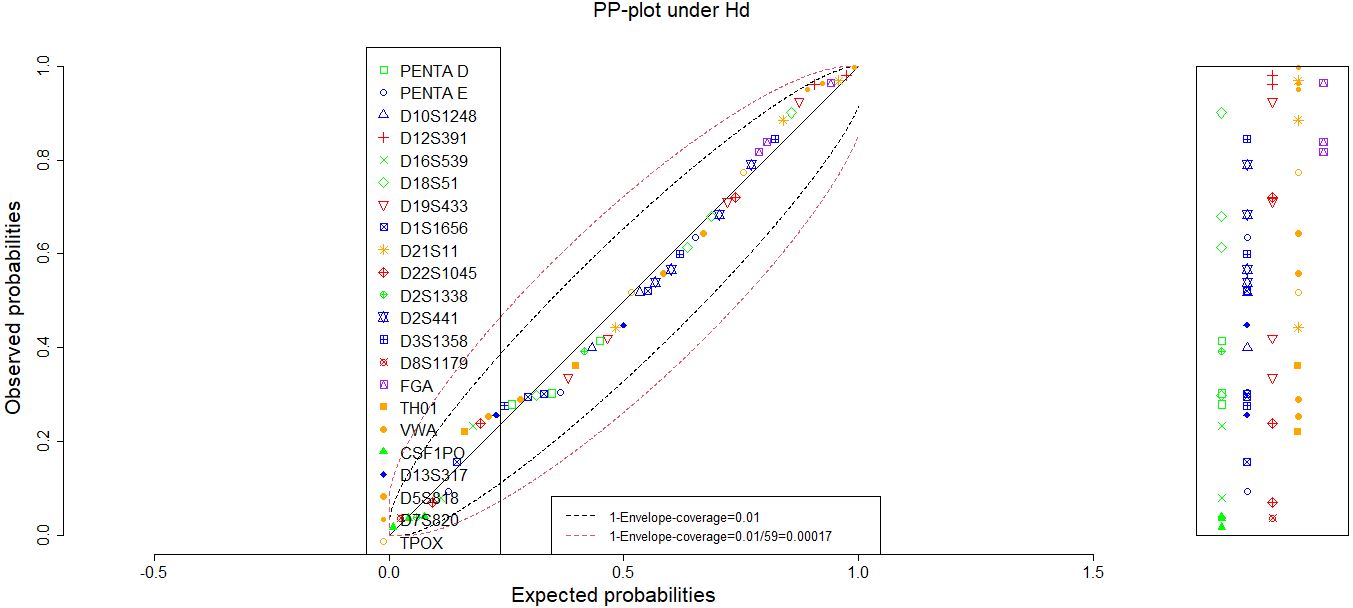  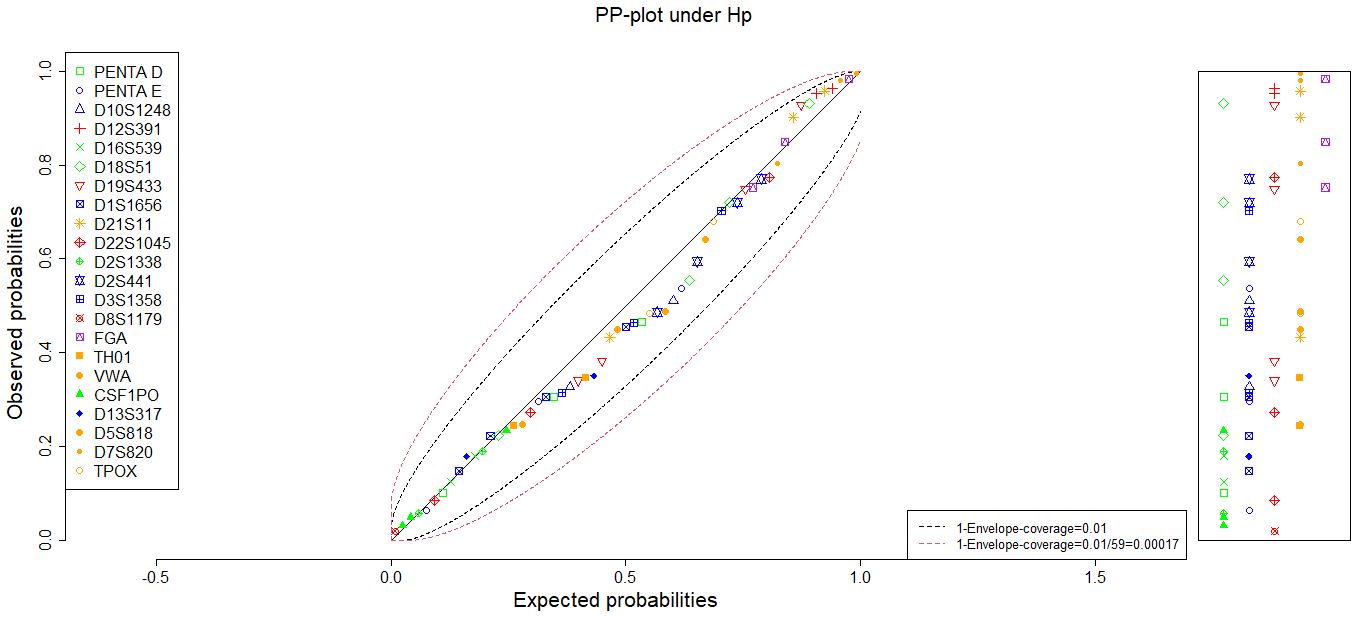 |
| --- |
| Q3  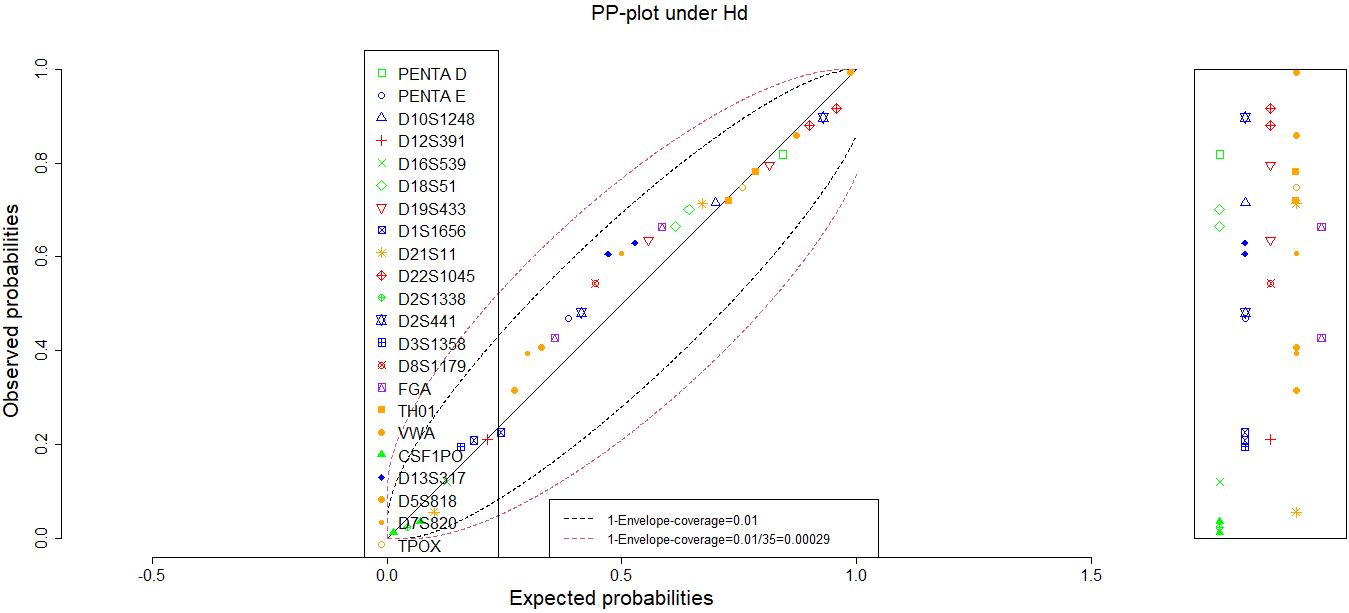  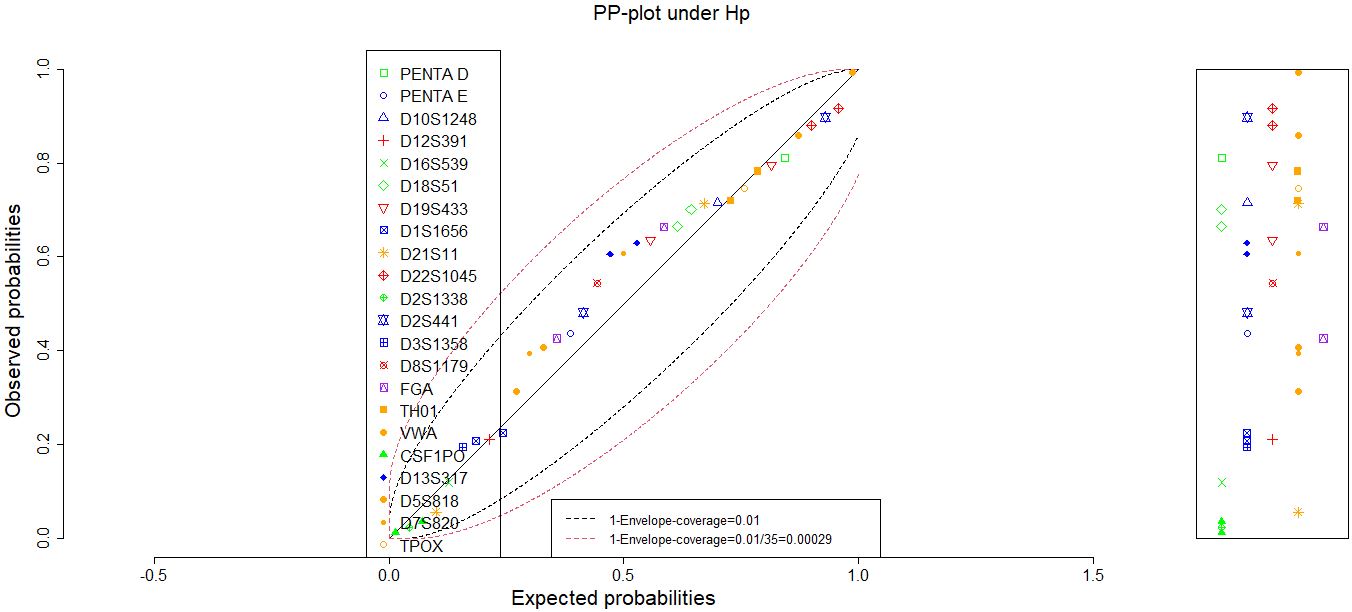 |
| Figure 3: Model validations for crime stains Q1 and Q3 were performed considering H_d_ (on top) and H_p_ (bellow), with a significance level of 0.01. |

| \|  \| **Case1Q4** \| \| **Case1Q5** \| \| \| --- \| --- \| --- \| --- \| --- \| \| **Locus** \| **EuroForMix**  **Major Genotype** \| **GeneMapper ID-X**  **Major Genotype** \| **EuroForMix**  **Major Genotype** \| **GeneMapper ID-X**  **Major Genotype** \| \| AMEL \| not considered* \| X,Y* \| not considered* \| X,Y* \| \| D10S1248 \| 14,16 \| 14,16 \| 14,16 \| 14,16 \| \| D12S391 \| 15,16 \| 15,16 \| 15,16 \| 15,16 \| \| D16S539 \| 9,11 \| 9,11 \| 9,11 \| 9,11 \| \| D18S51 \| 15,21 \| 15,21 \| 15,21 \| 15,21 \| \| D19S433 \| 14,14 \| 14,14 \| 14,14 \| 14,14 \| \| D1S1656 \| 16,16 \| 16,16 \| 16,16 \| 16,16 \| \| D21S11 \| 30,30 \| 30,30 \| 30,30 \| 30,30 \| \| D22S1045 \| 15,17 \| 15,17 \| 15,17 \| 15,17 \| \| D2S1338 \| 18,25 \| 18,25 \| 18,25 \| 18,25 \| \| D2S441 \| 12,15 \| 12,15 \| 12,15 \| 12,15 \| \| D3S1358 \| 15,17 \| 15,17 \| 15,17 \| 15,17 \| \| D8S1179 \| 14,14 \| 14,14 \| 14,14 \| 14,14 \| \| FGA \| 19,27* \| Inconclusive* \| 19,27 \| 19,27 \| \| TH01 \| 7,9.3 \| 7,9.3 \| 7,9.3 \| 7,9.3 \| \| VWA \| 14,17 \| 14,17 \| 14,17 \| 14,17 \| \| CSF1PO \| 10,12 \| 10,12 \| 10,12 \| 10,12 \| \| D13S317 \| 11,11 \| 11,11 \| 11,11 \| 11,11 \| \| D5S818 \| 11,12 \| 11,12 \| 11,12 \| 11,12 \| \| D7S820 \| 9,10 \| 9,10 \| 9,10 \| 9,10 \| \| TPOX \| 8,8 \| 8,8 \| 8,8 \| 8,8 \| \| SE33 \| not considered* \| not considered* \| not considered* \| not considered* \| \| Penta E \| 12,14 \| 12,14 \| 12,14 \| 12,14 \| \| Penta D \| 11,13 \| 11,13 \| 11,13 \| 11,13 \| |
| --- | --- | --- | --- | --- | --- | --- | --- | --- | --- | --- | --- | --- | --- | --- | --- | --- | --- | --- | --- | --- | --- | --- | --- | --- | --- | --- | --- | --- | --- | --- | --- | --- | --- | --- | --- | --- | --- | --- | --- | --- | --- | --- | --- | --- | --- | --- | --- | --- | --- | --- | --- | --- | --- | --- | --- | --- | --- | --- | --- | --- | --- | --- | --- | --- | --- | --- | --- | --- | --- | --- | --- | --- | --- | --- | --- | --- | --- | --- | --- | --- | --- | --- | --- | --- | --- | --- | --- | --- | --- | --- | --- | --- | --- | --- | --- | --- | --- | --- | --- | --- | --- | --- | --- | --- | --- | --- | --- | --- | --- | --- | --- | --- | --- | --- | --- | --- | --- | --- | --- | --- | --- | --- | --- | --- | --- | --- | --- | --- | --- | --- |
| Figure 4: Comparison of the major contributor deconvolution using EuroForMix and GeneMapper^TM^ ID-X of crime stain Q4 (left) and Q5 (right). There were agreement for the major points. The markers not considered or inconclusive in one of them are flagged with an asterisk (*). There were no points of discrepancy. |

Case 2

| \| Vehicle 1  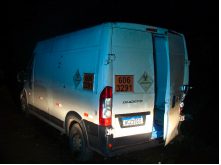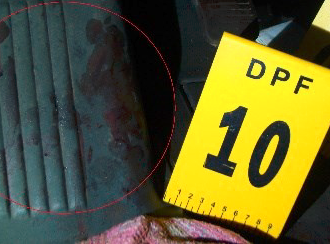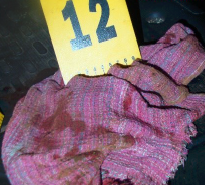 \| \| --- \| \| Vehicle 2  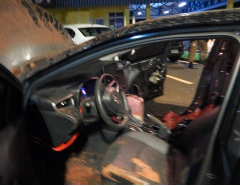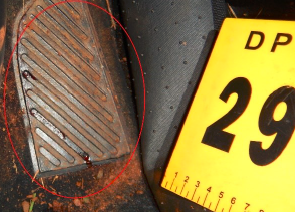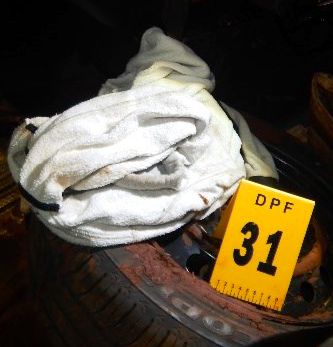 \| \| Vehicle 3  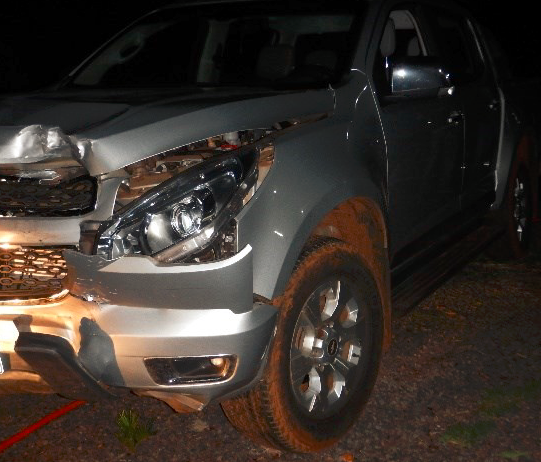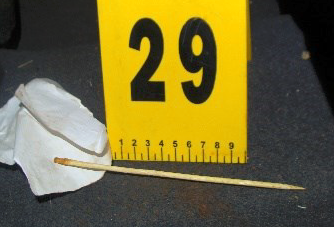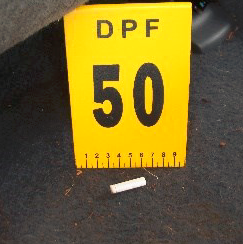 \| |
| --- | --- | --- | --- |
| Figure 5. Crime scene forensic analyses photos taken by KÜHL, L.A. and ANJOS, D.S. on March 25, 2022. |

| CASE 2  Q1  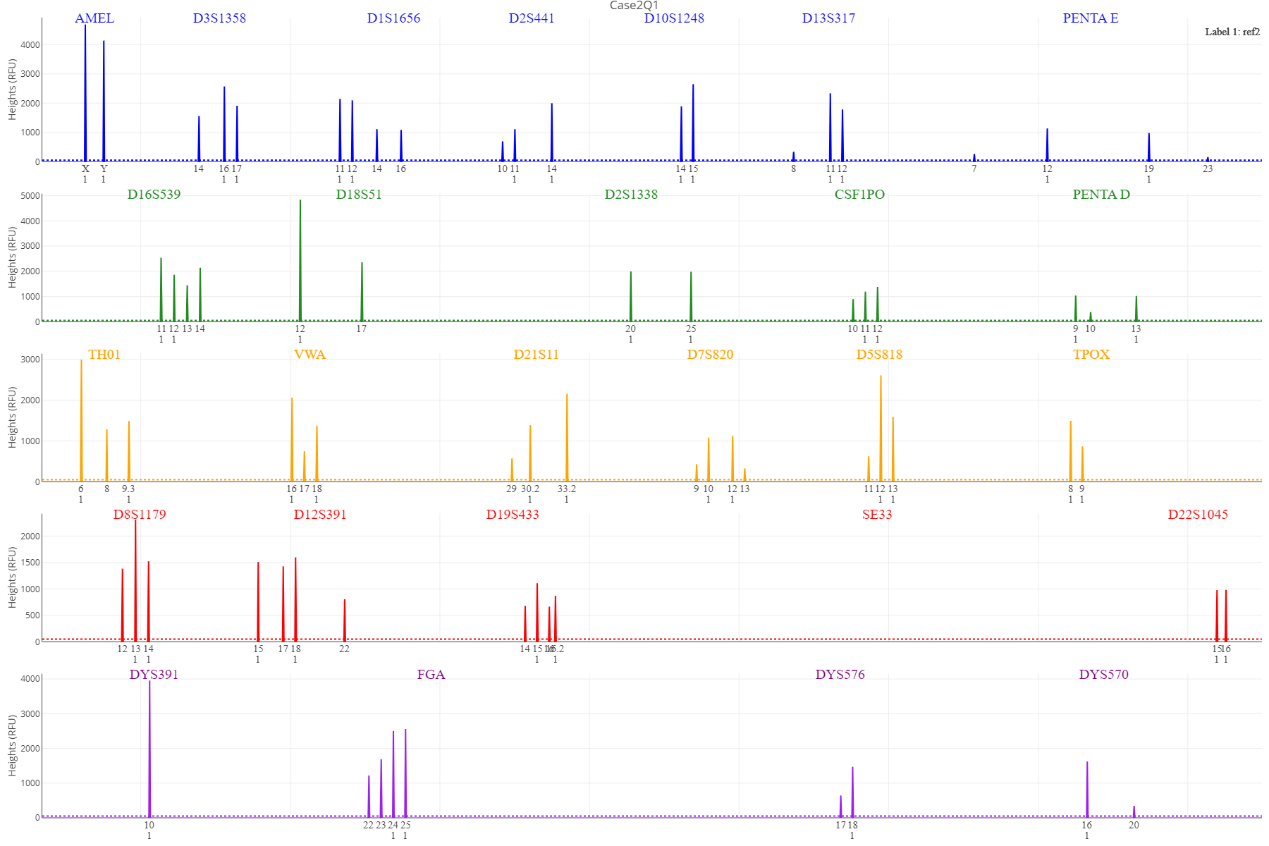 |
| --- |
| Q3  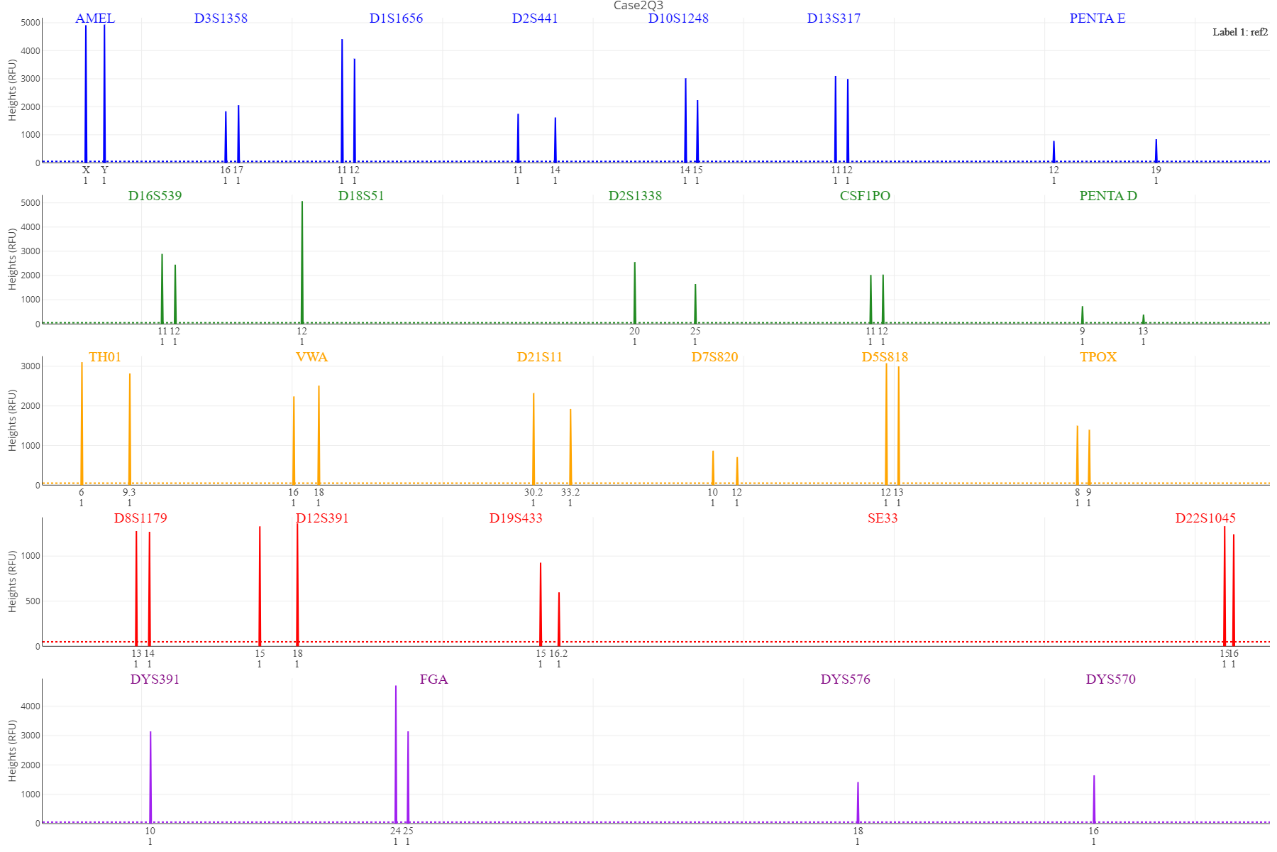 |
| Q6  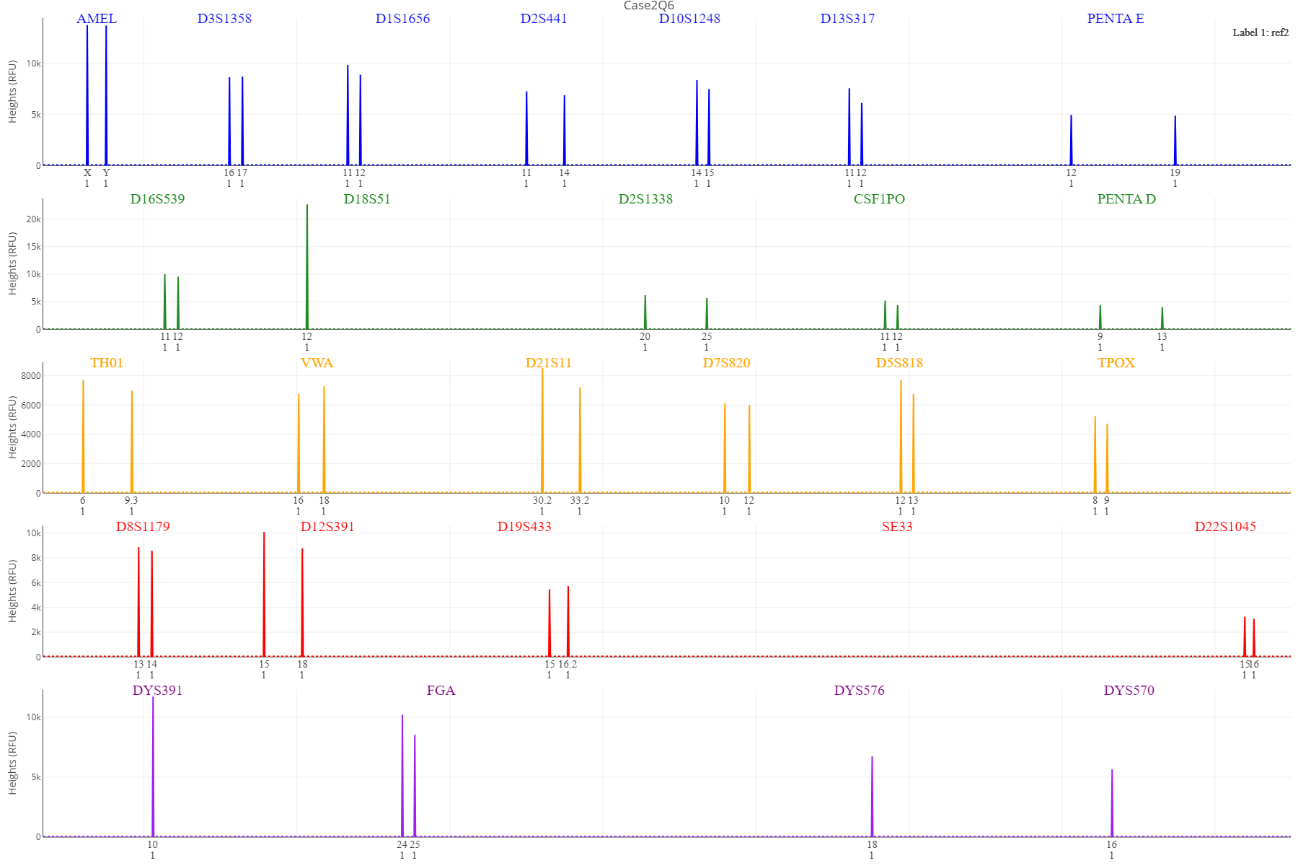 |
| Q7  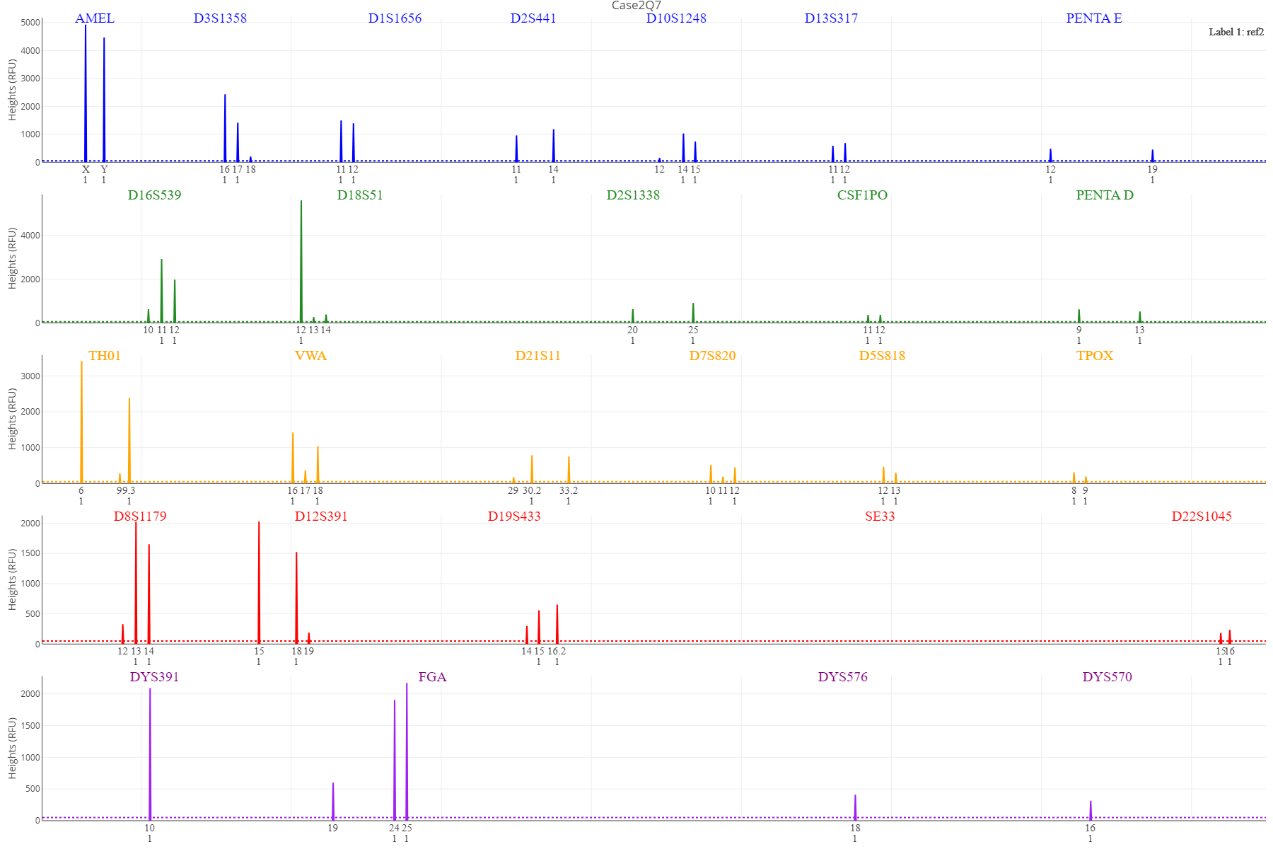 |
| Q18  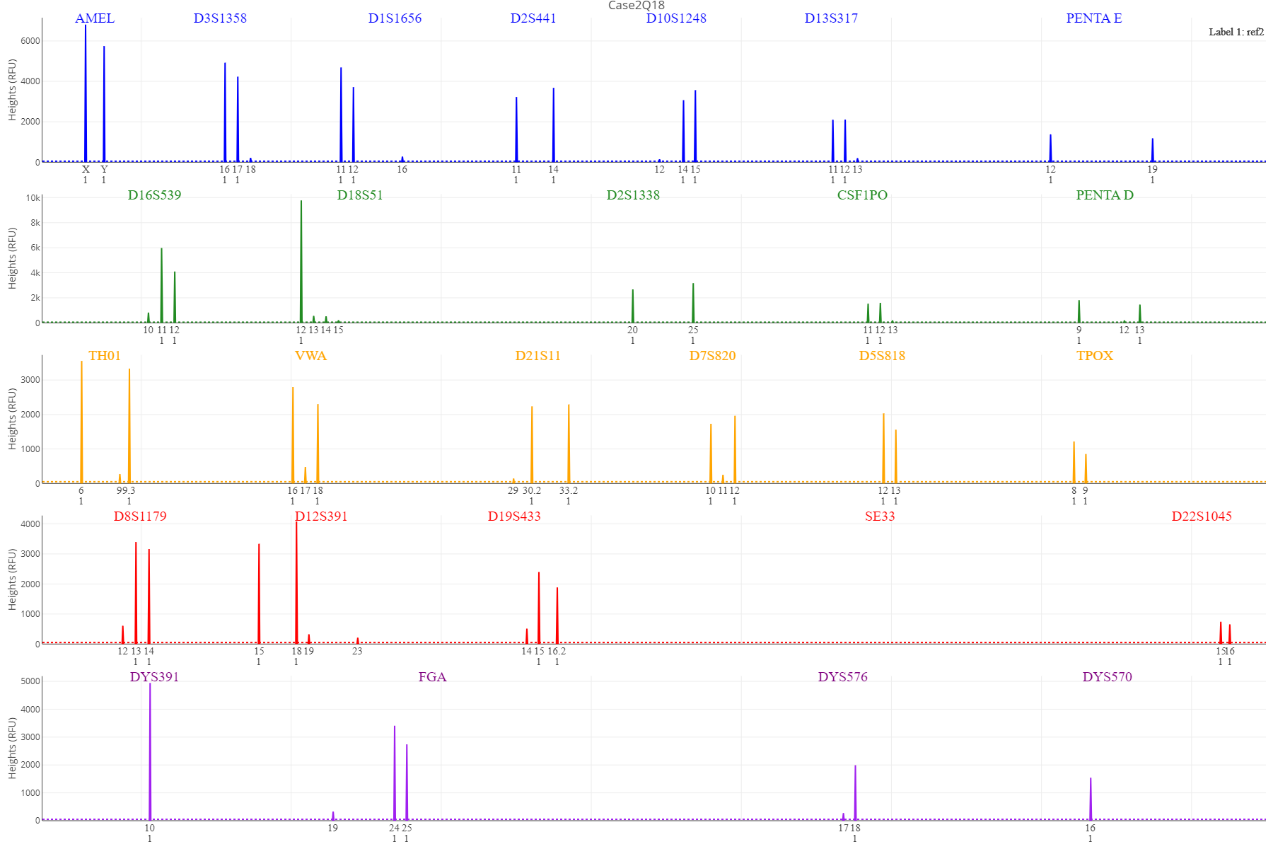 |
| Q19  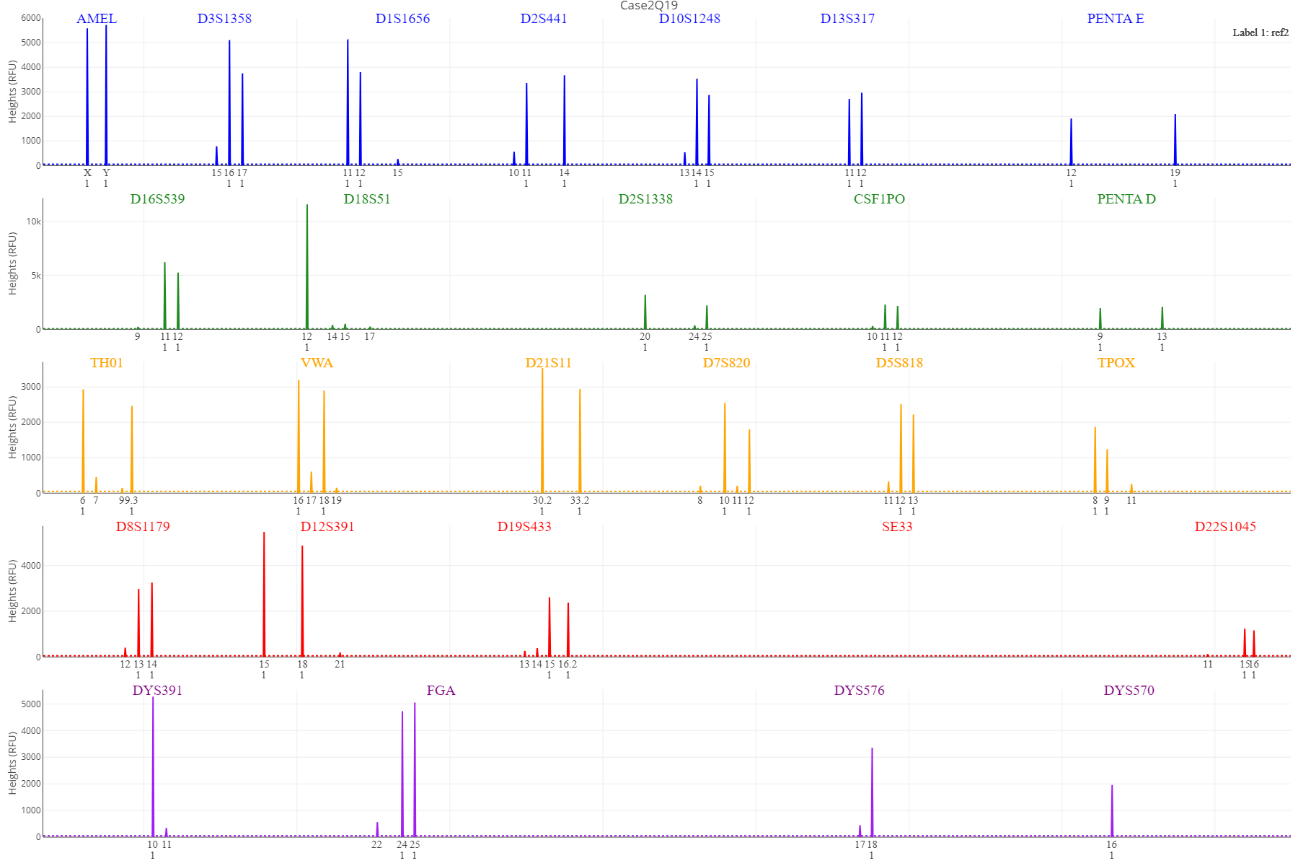 |
| Q15  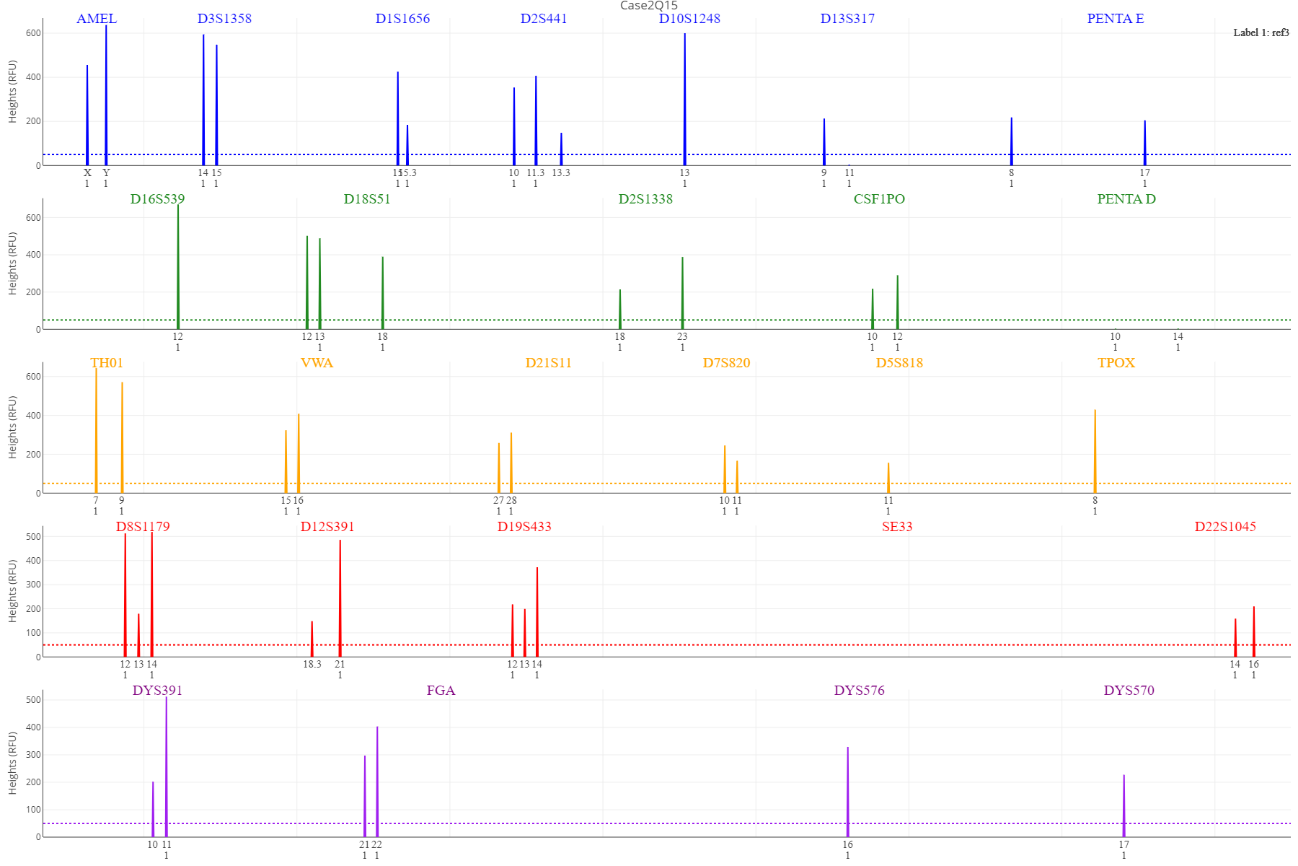 |
| Q29  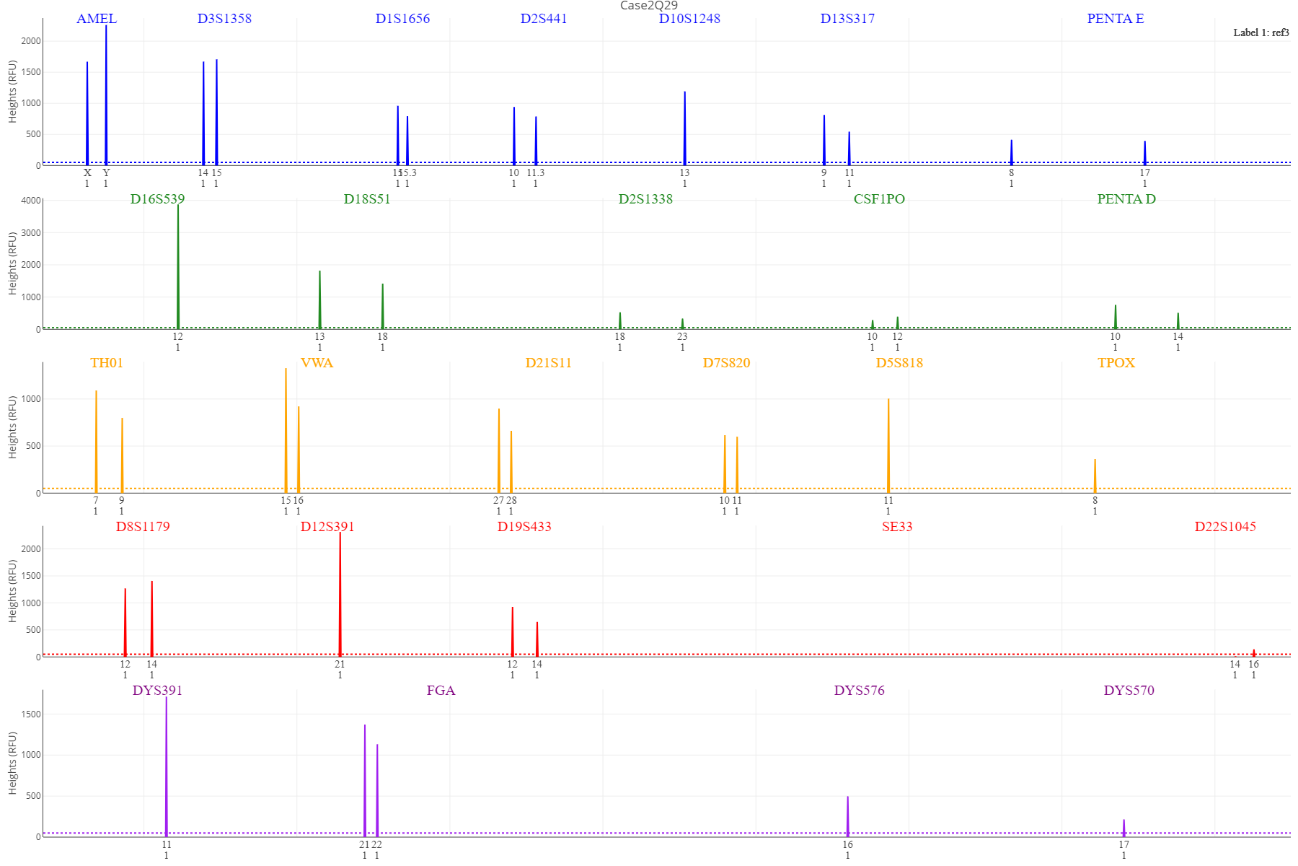 |
| Figure 6. The allele and peak height representations of Case 2 crime stains. The reference ref2 is labeled with the number "1" in Q1, Q3, Q6, Q7, Q18 and Q19; and the ref3 is labeled with number “1” in Q15 and Q29. |

| CASE 2  Q1  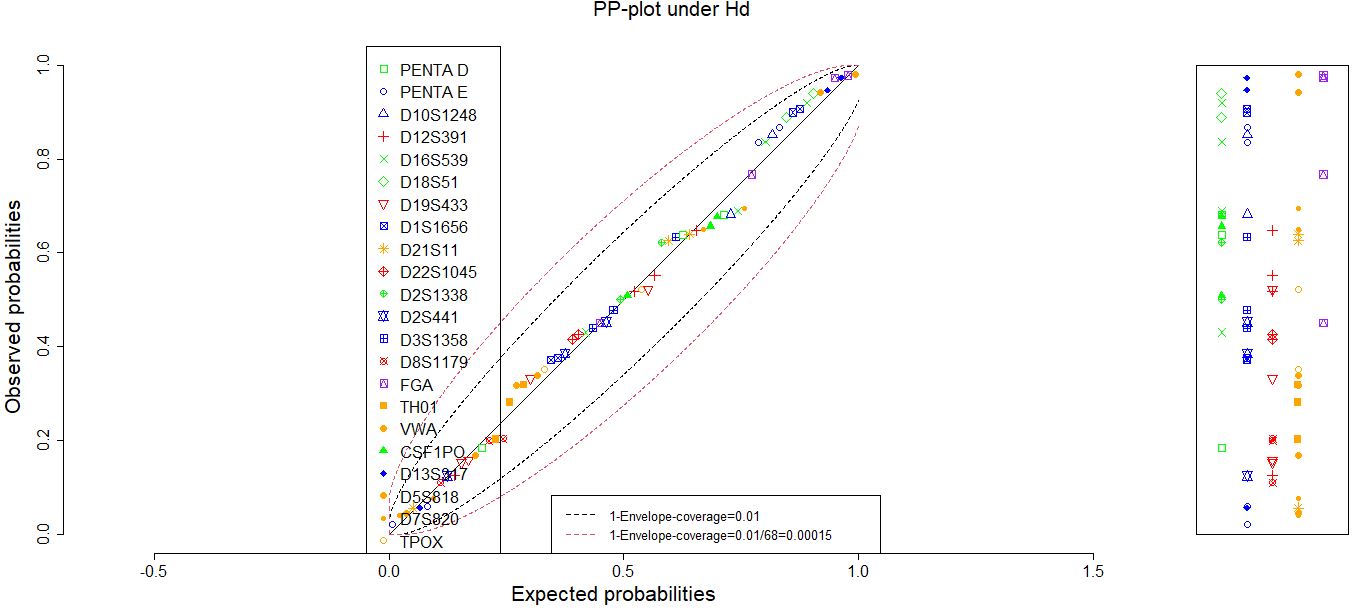  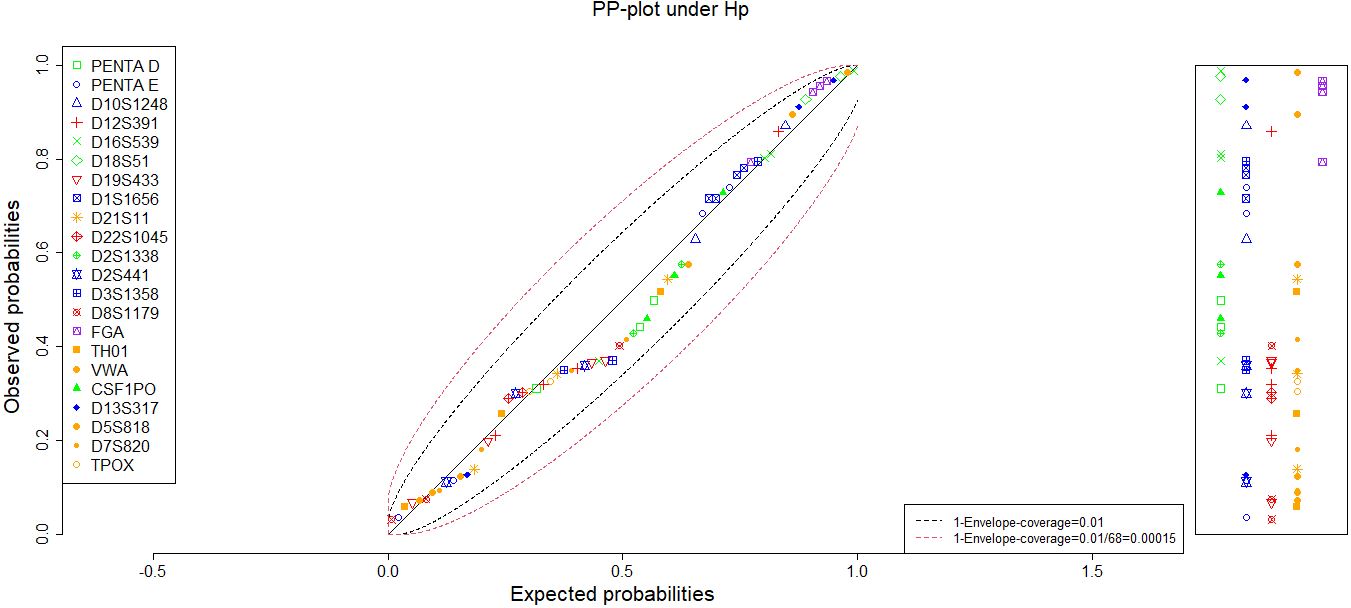 |
| --- |
| Q3  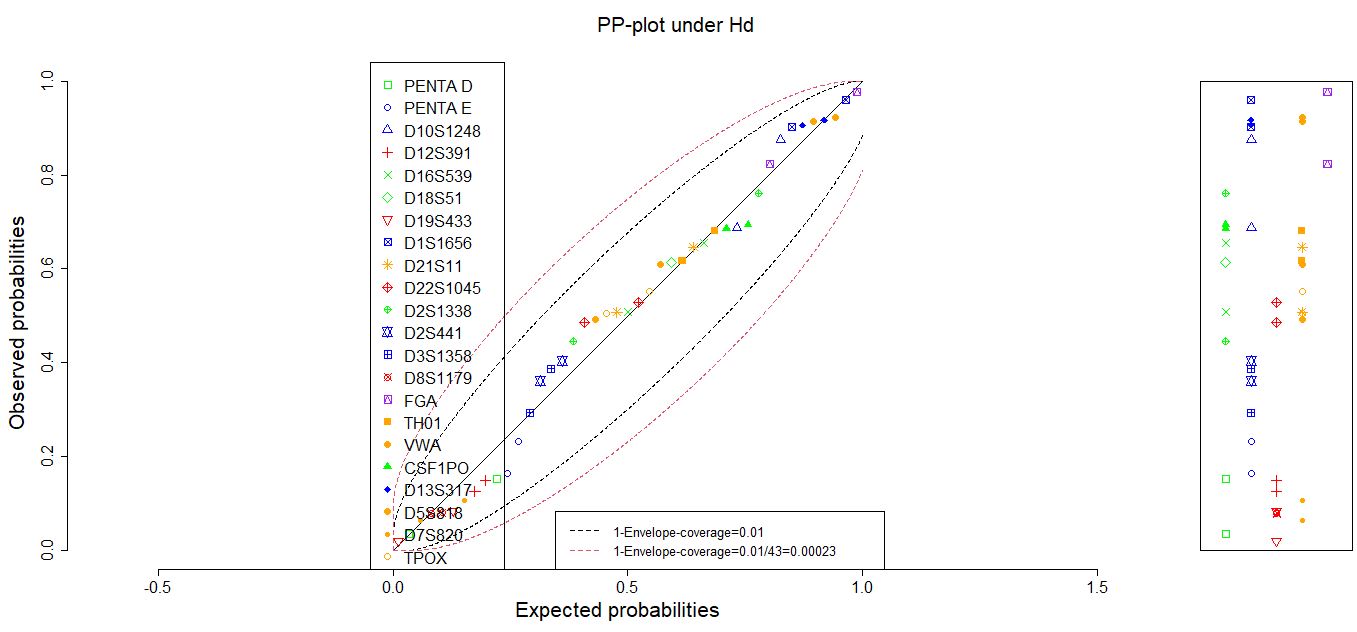  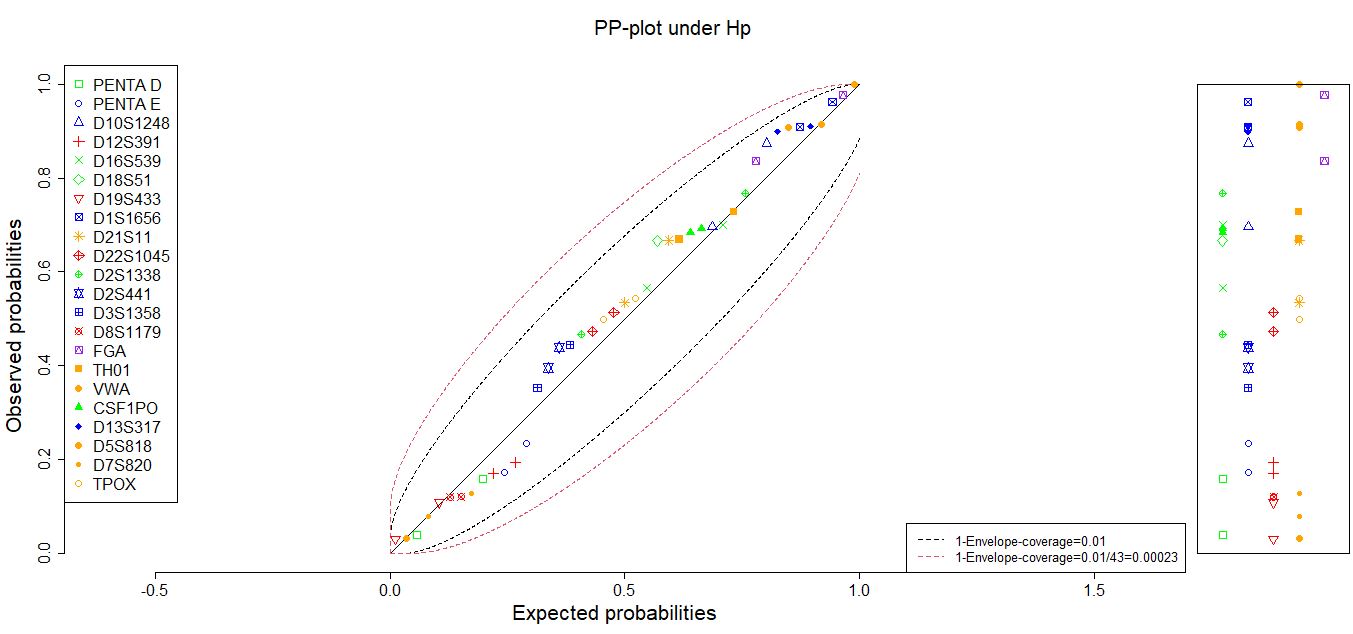 |
| Q6  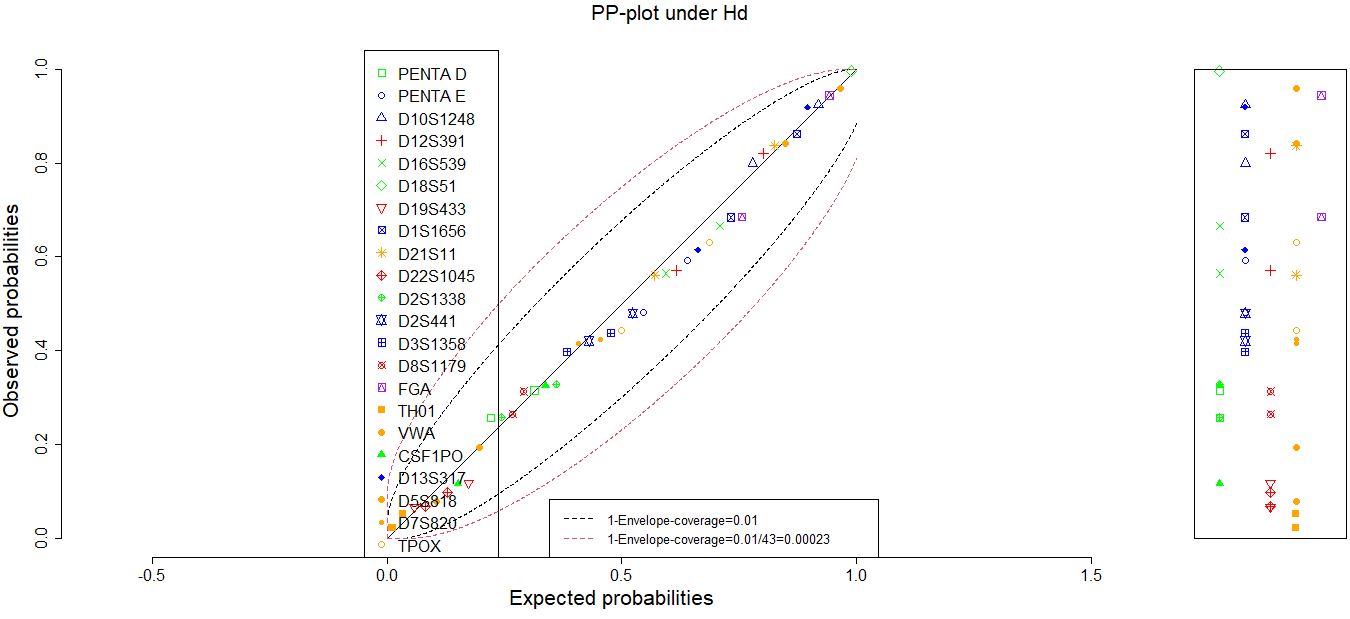  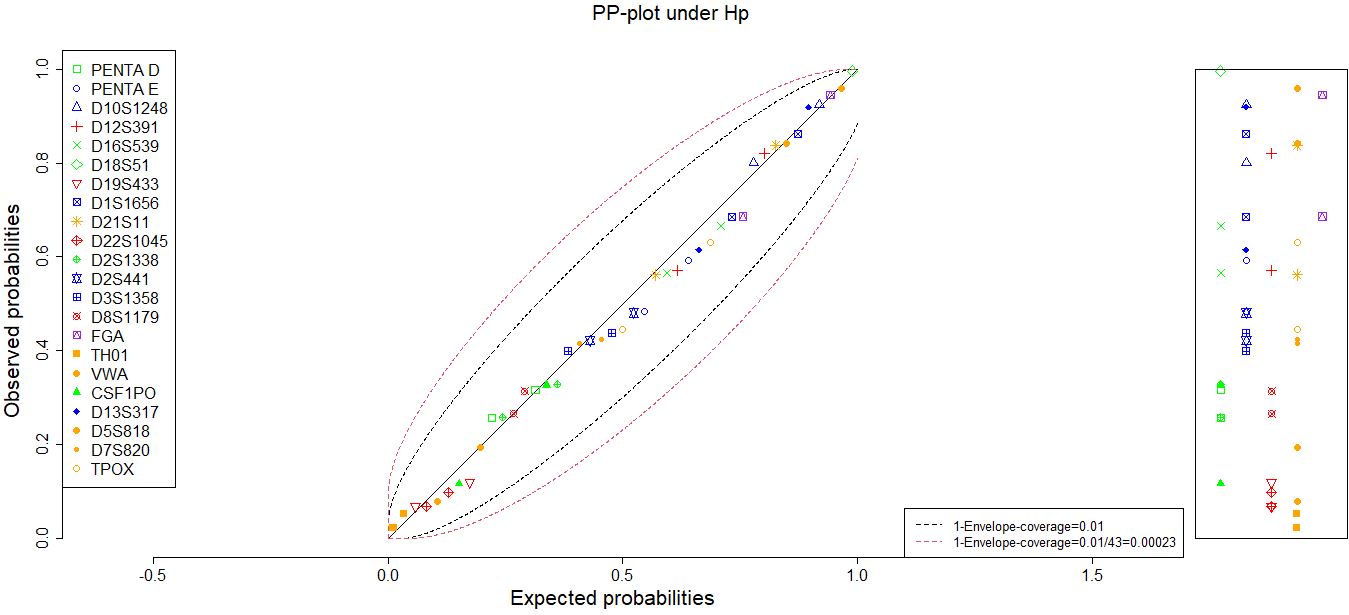 |
| Q7  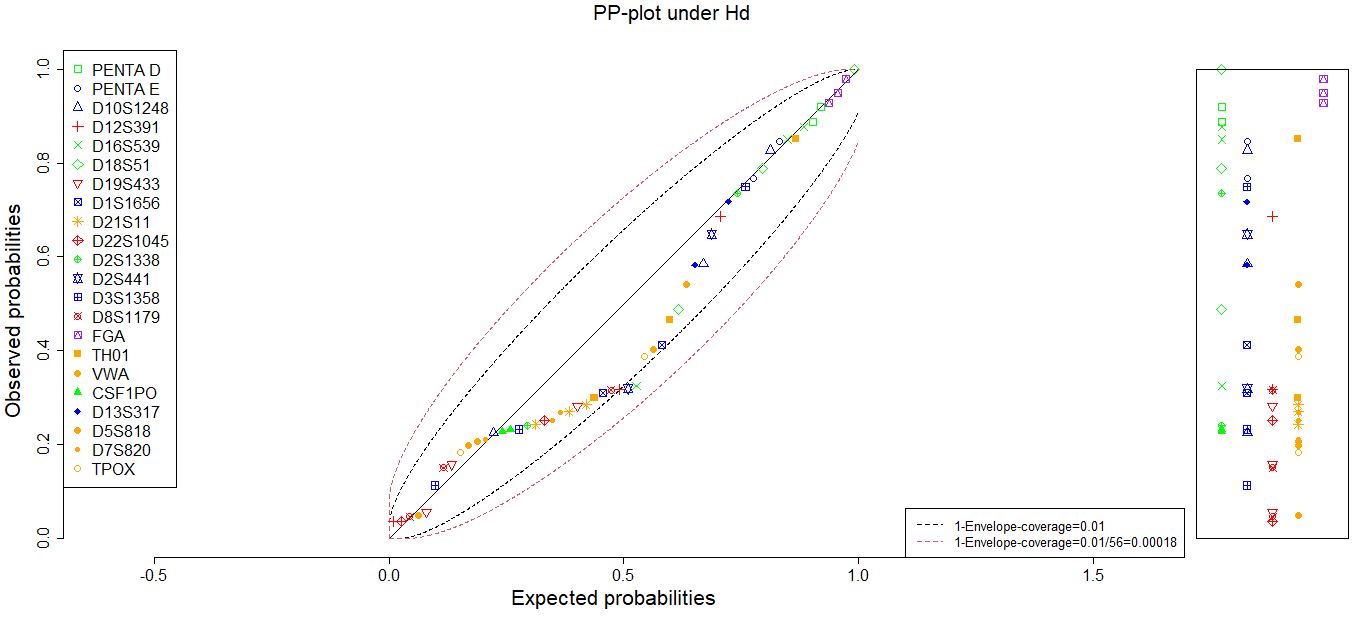  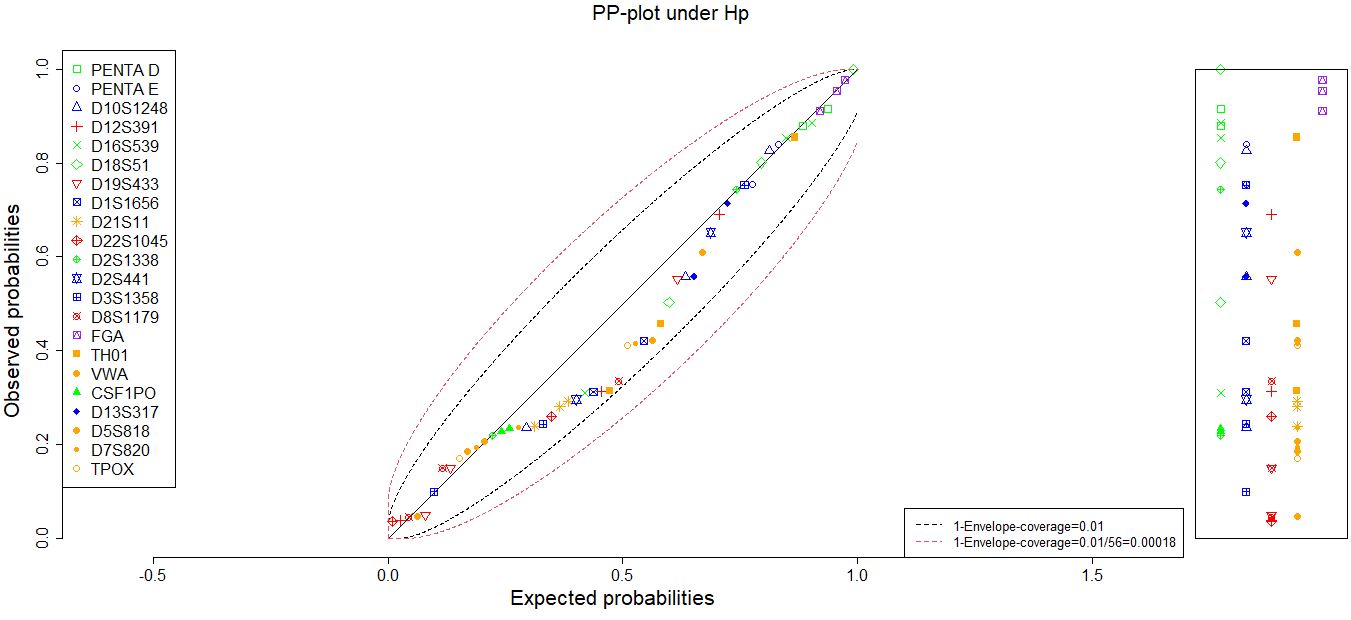 |
| Q18  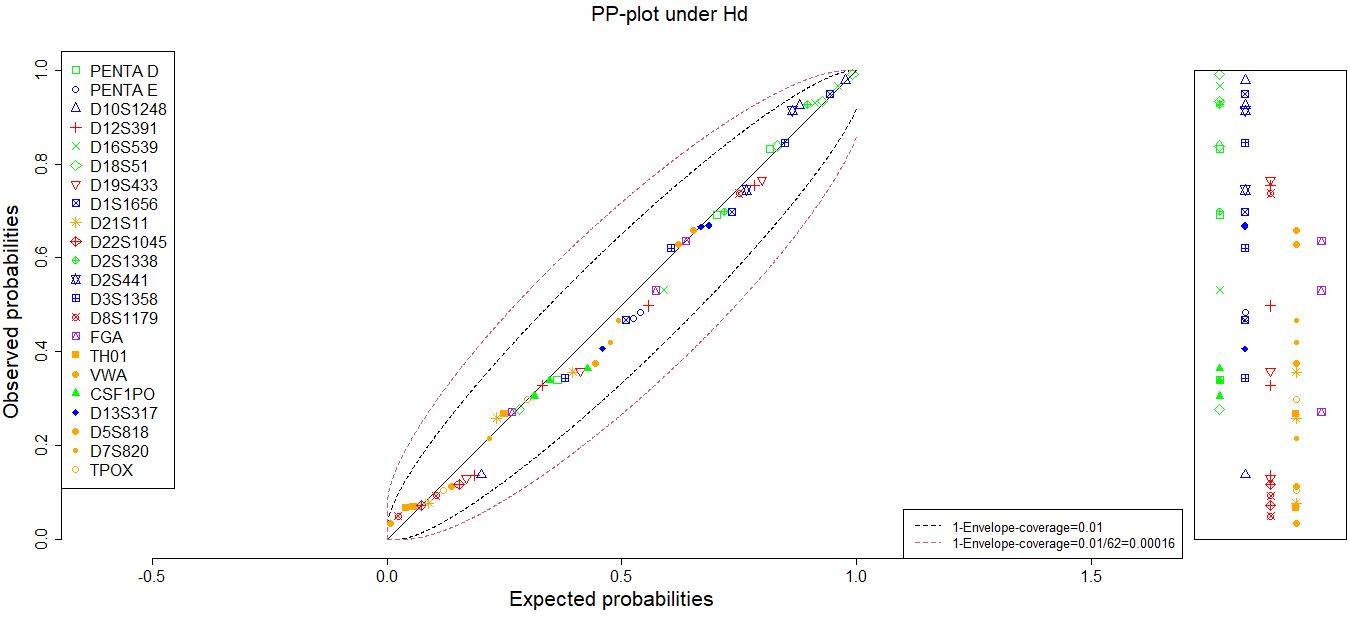  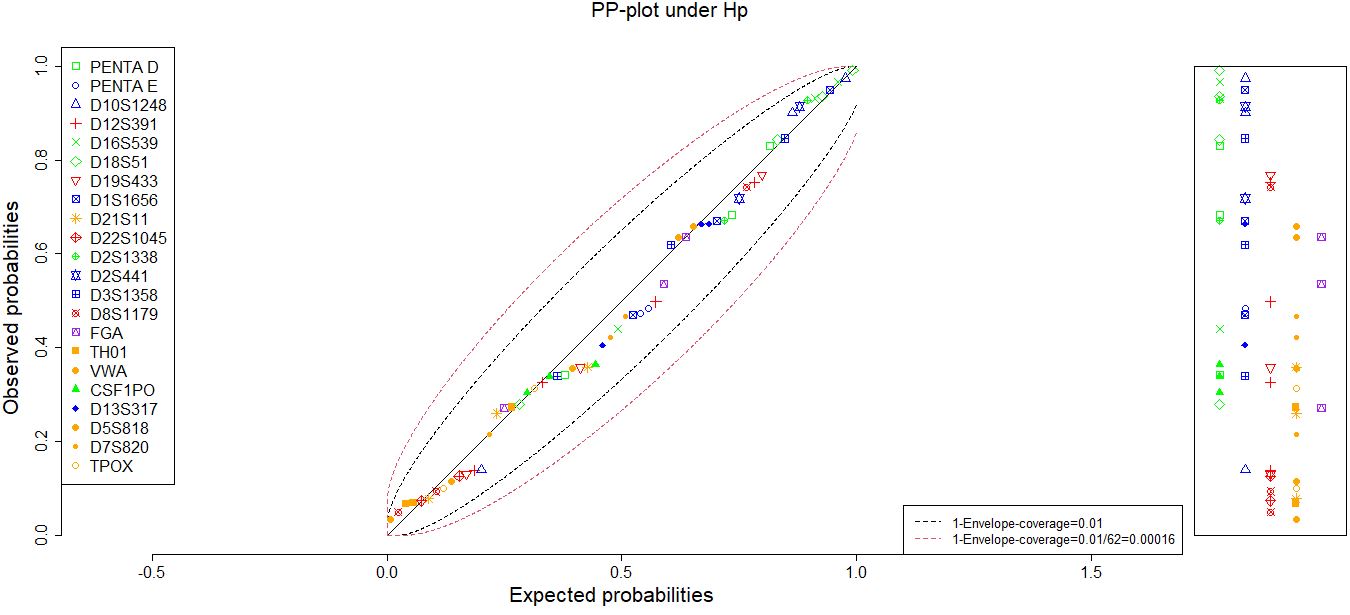 |
| Q19  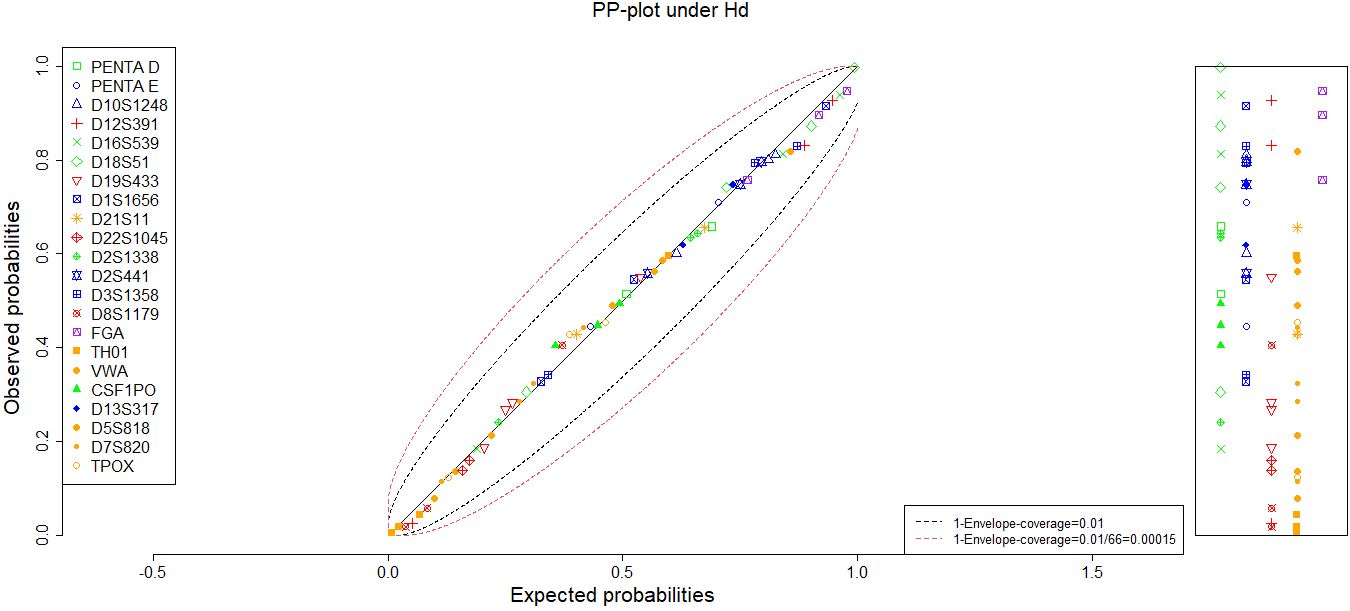  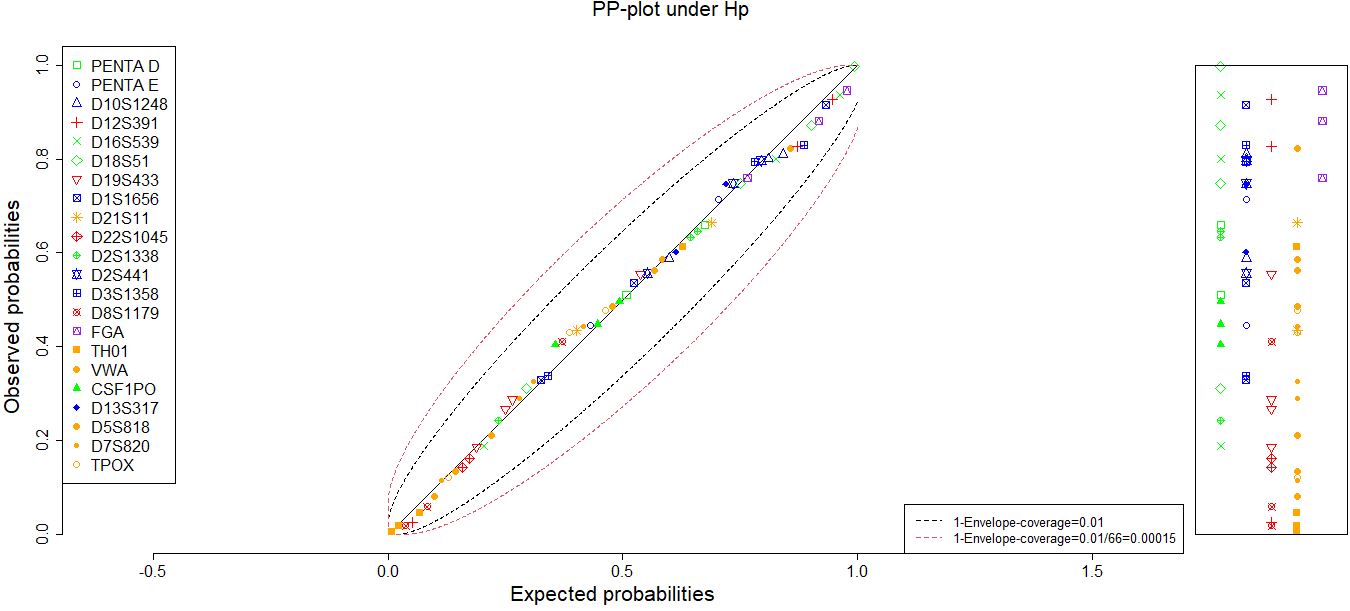 |
| Q15  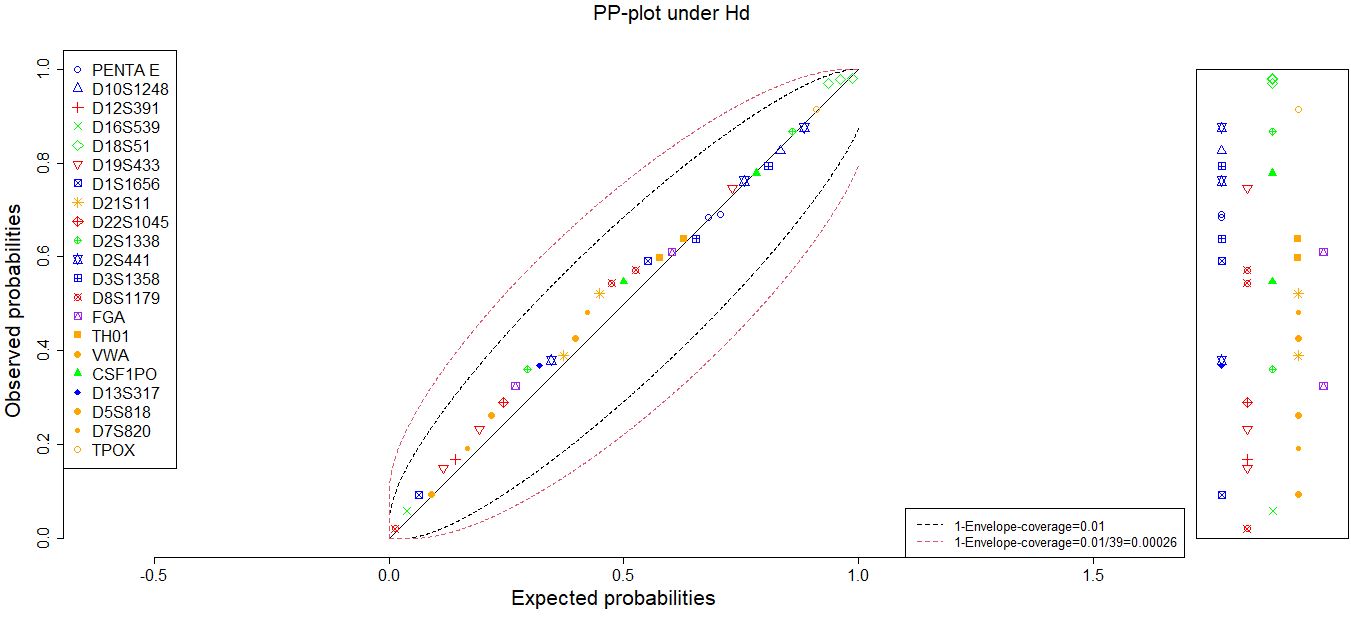  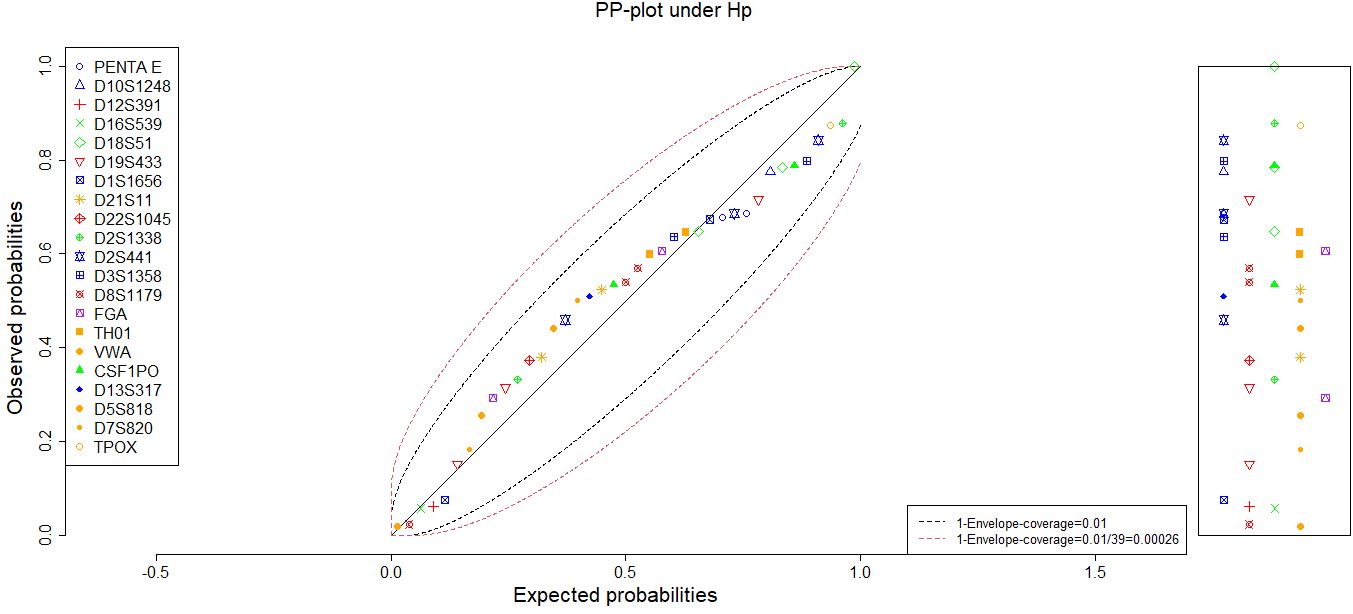 |
| Q29  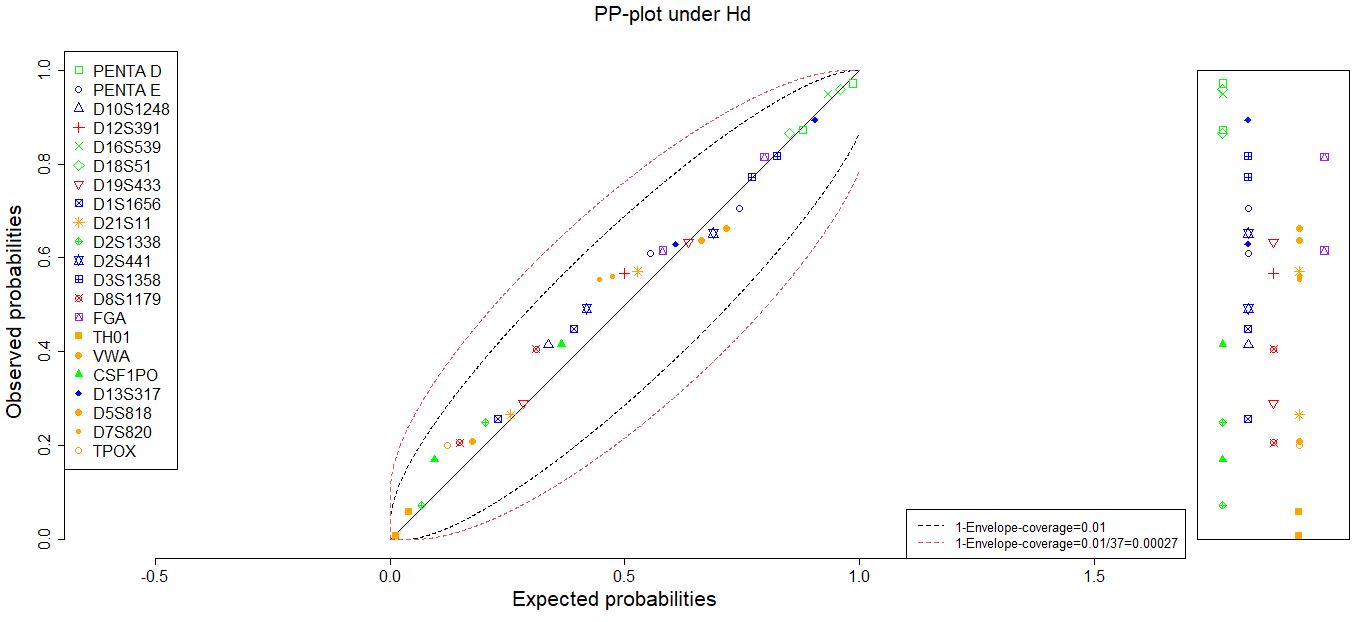  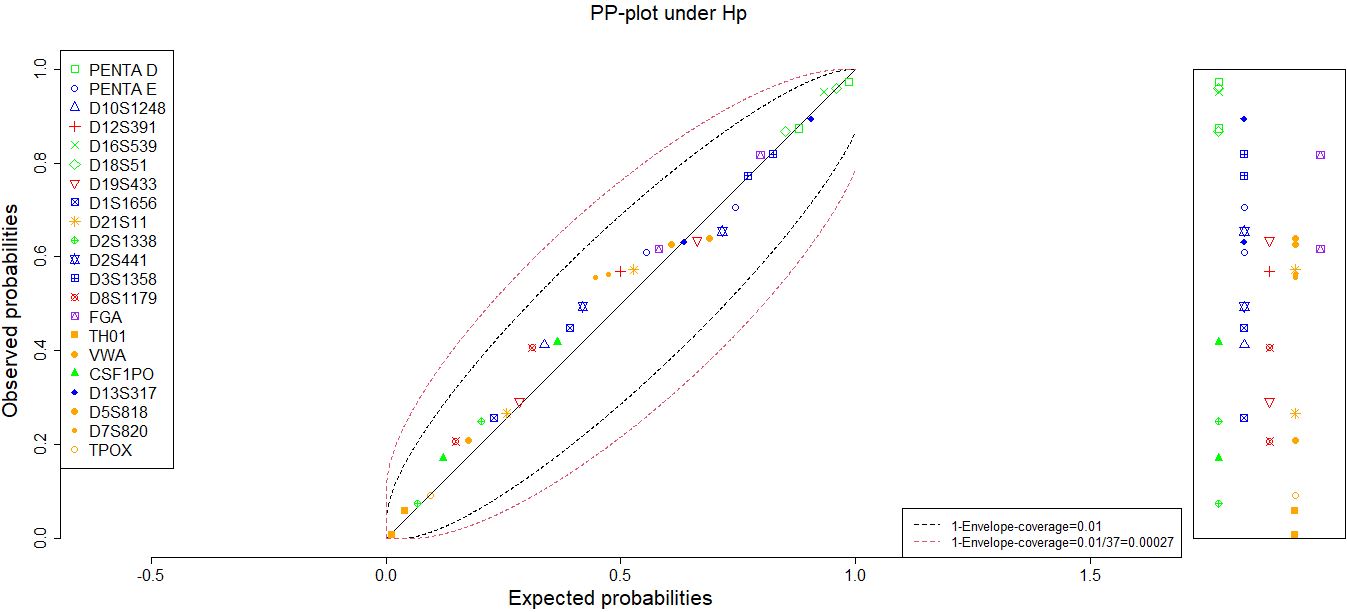 |
| Figure 7: Model validation for crime stains Q1, Q3, Q6, Q7, Q18, Q19, Q15 e Q29 was performed considering H_d_ (on top) and H_p_ (bellow), with a significance level of 0.01. |

| \|  \| **Case2Q1** \| \| **Case2Q7** \| \| \| --- \| --- \| --- \| --- \| --- \| \| **Locus** \| **EuroForMix**  **Major Genotype** \| **GeneMapper ID-X**  **Major Genotype** \| **EuroForMix**  **Major Genotype** \| **GeneMapper ID-X**  **Major Genotype** \| \| AMEL \| not considered* \| Inconclusive* \| not considered* \| X,Y* \| \| D10S1248 \| Inconclusive \| Inconclusive \| 14,15 \| 14,15 \| \| D12S391 \| Inconclusive \| Inconclusive \| 15,18* \| Inconclusive* \| \| D16S539 \| Inconclusive \| Inconclusive \| 11,12 \| 11,12 \| \| D18S51 \| Inconclusive \| Inconclusive \| 12,12* \| Inconclusive* \| \| D19S433 \| Inconclusive \| Inconclusive \| 15,16.2 \| 15,16.2 \| \| D1S1656 \| Inconclusive* \| 11,12* \| 11,12 \| 11,12 \| \| D21S11 \| Inconclusive* \| 30.2,33.2* \| 30.2,33.2 \| 30.2,33.2 \| \| D22S1045 \| Inconclusive* \| 15,16* \| Inconclusive* \| 15,16* \| \| D2S1338 \| Inconclusive* \| 20,25* \| 20,25 \| 20,25 \| \| D2S441 \| Inconclusive* \| 11,14* \| 11,14 \| 11,14 \| \| D3S1358 \| Inconclusive \| Inconclusive \| 16,17* \| Inconclusive* \| \| D8S1179 \| Inconclusive* \| 12,14* \| 13,14 \| 13,14 \| \| FGA \| Inconclusive \| Inconclusive \| 24,25 \| 24,25 \| \| TH01 \| Inconclusive \| Inconclusive \| 6,9.3* \| Inconclusive* \| \| VWA \| Inconclusive* \| 16,18* \| 16,18 \| 16,18 \| \| CSF1PO \| Inconclusive \| Inconclusive \| 11,12 \| 11,12 \| \| D13S317 \| Inconclusive* \| 11,12* \| 11,12 \| 11,12 \| \| D5S818 \| Inconclusive * \| 12,13* \| 12,13 \| 12,13 \| \| D7S820 \| Inconclusive \| Inconclusive \| 10,12 \| 10,12 \| \| TPOX \| Inconclusive \| Inconclusive \| Inconclusive* \| 8,9* \| \| SE33 \| not considered \| not considered \| not considered \| not considered \| \| Penta E \| Inconclusive \| Inconclusive \| 12,19 \| 12,19 \| \| Penta D \| Inconclusive \| Inconclusive \| 9,13 \| 9,13 \| |
| --- | --- | --- | --- | --- | --- | --- | --- | --- | --- | --- | --- | --- | --- | --- | --- | --- | --- | --- | --- | --- | --- | --- | --- | --- | --- | --- | --- | --- | --- | --- | --- | --- | --- | --- | --- | --- | --- | --- | --- | --- | --- | --- | --- | --- | --- | --- | --- | --- | --- | --- | --- | --- | --- | --- | --- | --- | --- | --- | --- | --- | --- | --- | --- | --- | --- | --- | --- | --- | --- | --- | --- | --- | --- | --- | --- | --- | --- | --- | --- | --- | --- | --- | --- | --- | --- | --- | --- | --- | --- | --- | --- | --- | --- | --- | --- | --- | --- | --- | --- | --- | --- | --- | --- | --- | --- | --- | --- | --- | --- | --- | --- | --- | --- | --- | --- | --- | --- | --- | --- | --- | --- | --- | --- | --- | --- | --- | --- | --- | --- | --- |
| \|  \| **Case2Q18** \| \| **Case2Q19** \| \| \| --- \| --- \| --- \| --- \| --- \| \| **Locus** \| **EuroForMix**  **Major Genotype** \| **GeneMapper ID-X**  **Major Genotype** \| **EuroForMix**  **Major Genotype** \| **GeneMapper ID-X**  **Major Genotype** \| \| AMEL \| not considered* \| X,Y* \| not considered* \| X,Y* \| \| D10S1248 \| 14,15 \| 14,15 \| 14,15 \| 14,15 \| \| D12S391 \| 15,18* \| Inconclusive* \| 15,18 \| 15,18 \| \| D16S539 \| 11,12* \| Inconclusive* \| 11,12* \| Inconclusive* \| \| D18S51 \| 12,12* \| Inconclusive* \| 12,12* \| Inconclusive* \| \| D19S433 \| 15,16.2 \| 15,16.28 \| 15,16.2* \| Inconclusive* \| \| D1S1656 \| 11,12* \| Inconclusive \| 11,12* \| Inconclusive* \| \| D21S11 \| 30.2,33.2 \| 30.2,33.2 \| 30.2,33.2 \| 30.2,33.2 \| \| D22S1045 \| 15,16 \| 15,16 \| 15,16 \| 15,16 \| \| D2S1338 \| 20,25 \| 20,25 \| 20,25* \| Inconclusive* \| \| D2S441 \| 11,14 \| 11,14 \| 11,14 \| 11,14 \| \| D3S1358 \| 16,17 \| 16,17 \| 16,17 \| 16,17 \| \| D8S1179 \| 13,14 \| 13,14 \| 13,14 \| 13,14 \| \| FGA \| 24,25 \| 24,25 \| 24,25 \| 24,25 \| \| TH01 \| 6,9.3 \| 6,9.3 \| 6,9.3* \| Inconclusive* \| \| VWA \| 16,18 \| 16,18 \| 16,18* \| Inconclusive* \| \| CSF1PO \| 11,12 \| 11,12 \| 11,12 \| 11,12 \| \| D13S317 \| 11,12 \| 11,12 \| 11,12 \| 11,12 \| \| D5S818 \| 12,13 \| 12,13 \| 12,13 \| 12,13 \| \| D7S820 \| 10,12 \| 10,12 \| 10,12* \| Inconclusive* \| \| TPOX \| 8,9 \| 8,9 \| 8,9* \| Inconclusive* \| \| SE33 \| not considered \| not considered \| not considered \| not considered \| \| Penta E \| 12,19 \| 12,19 \| 12,19 \| 12,19 \| \| Penta D \| 9,13 \| 9,13 \| 9,13 \| 9,13 \| |
| \|  \| **Case2Q15** \| \| \| --- \| --- \| --- \| \| **Locus** \| **EuroForMix**  **Major Genotype** \| **GeneMapper ID-X**  **Major Genotype** \| \| AMEL \| not considered* \| X,Y* \| \| D10S1248 \| 13,13 \| 13,13 \| \| D12S391 \| inconclusive \| Inconclusive \| \| D16S539 \| 12,12 \| 12,12 \| \| D18S51 \| inconclusive \| Inconclusive \| \| D19S433 \| inconclusive \| Inconclusive \| \| D1S1656 \| inconclusive \| Inconclusive \| \| D21S11 \| 27,28 \| 27,28 \| \| D22S1045 \| inconclusive \| Inconclusive \| \| D2S1338 \| inconclusive \| Inconclusive \| \| D2S441 \| Inconclusive* \| 10,11.3* \| \| D3S1358 \| 14,15 \| 14,15 \| \| D8S1179 \| 12,14 \| 12,14 \| \| FGA \| 21,22* \| Inconclusive* \| \| TH01 \| 7,9 \| 7,9 \| \| VWA \| 15,16* \| Inconclusive* \| \| CSF1PO \| inconclusive \| Inconclusive \| \| D13S317 \| inconclusive \| Inconclusive \| \| D5S818 \| inconclusive \| Inconclusive \| \| D7S820 \| inconclusive \| Inconclusive \| \| TPOX \| inconclusive \| Inconclusive \| \| SE33 \| not considered \| not considered \| \| Penta E \| Inconclusive* \| 8,17* \| \| Penta D \| inconclusive \| Inconclusive \| |
| Figure 8: Comparison of the major contributor deconvolution using EuroForMix and GeneMapper^TM^ ID-X of Case 2 crime stains Q1, Q7, Q18, Q19, Q15, and Q29. There were agreement for the major points and there were no discrepancies. The markers not considered or inconclusive in one of them are flagged with an asterisk (*). |
