# Supplementary figures and images for: Reanalyzing DNA mixture: a evaluation of EuroForMix for deconvolution and weight-of-evidence computing
Source: Forensic Sci Med Pathol. 2025 Oct 2;21(4):1813–9. doi: 10.1007/s12024-024-00872-x (PMC12799628; doi:10.1007/s12024-024-00872-x)

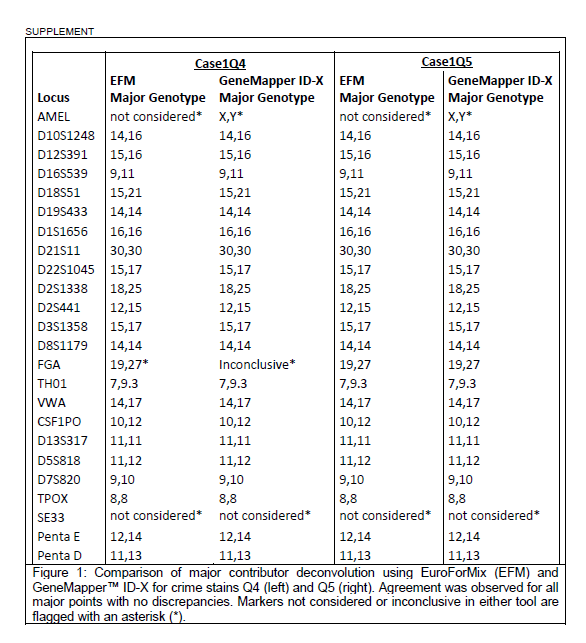


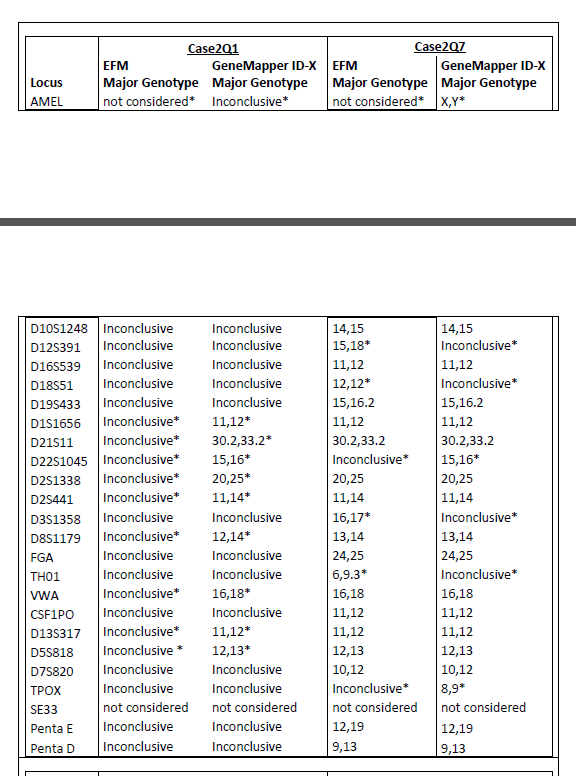


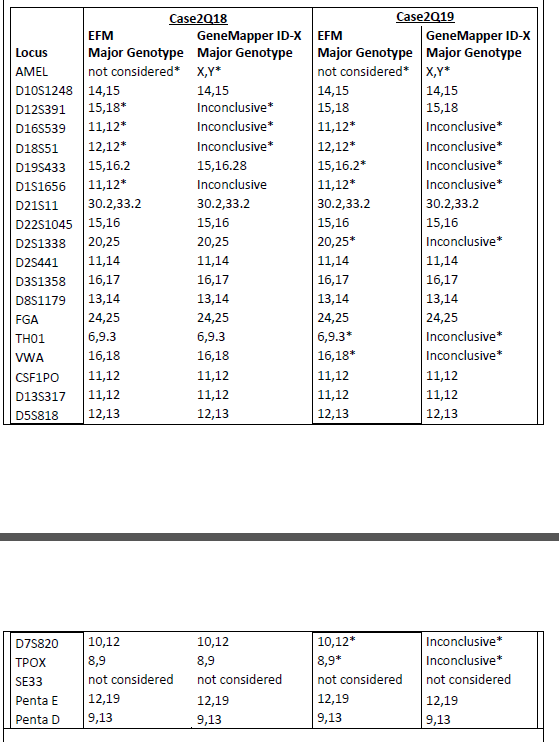


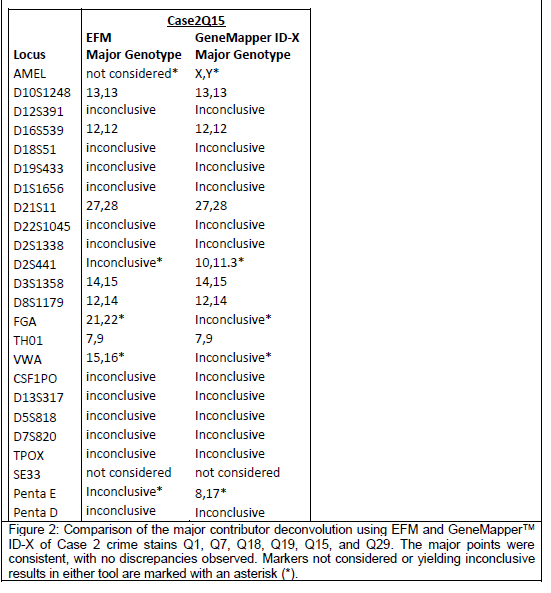

Supplement: Supplementary file 2 — Supplementary file2 (DOCX 6236 KB) [file 12024_2024_872_MOESM2_ESM.docx]
